# Performance Analysis of Distributed Estimation for Data Fusion Using a Statistical Approach in Smart Grid Noisy Wireless Sensor Networks

**DOI:** 10.3390/s20020567

**Published:** 2020-01-20

**Authors:** Chatura Seneviratne, Patikiri Arachchige Don Shehan Nilmantha Wijesekara, Henry Leung

**Affiliations:** 1 Department of Electrical and Information Engineering, Faculty of Engineering, University of Ruhuna, Galle 80000, Southern Province, Sri Lanka; chatura@eie.ruh.ac.lk; 2 Department of Electrical and Computer Engineering, Faculty of Information and Communication Technology, University of Calgary, Calgary, AB T5J0N3, Canada; leungh@ucalgary.ca

**Keywords:** data fusion, distributed estimation, energy efficiency, latency, fusion complexity, information accuracy, internet of things, smart grid communications

## Abstract

Internet of Things (IoT) can significantly enhance various aspects of today’s electric power grid infrastructures for making reliable, efficient, and safe next-generation Smart Grids (SGs). However, harsh and complex power grid infrastructures and environments reduce the accuracy of the information propagating through IoT platforms. In particularly, information is corrupted due to the measurement errors, quantization errors, and transmission errors. This leads to major system failures and instabilities in power grids. Redundant information measurements and retransmissions are traditionally used to eliminate the errors in noisy communication networks. However, these techniques consume excessive resources such as energy and channel capacity and increase network latency. Therefore, we propose a novel statistical information fusion method not only for structural chain and tree-based sensor networks, but also for unstructured bidirectional graph noisy wireless sensor networks in SG environments. We evaluate the accuracy, energy savings, fusion complexity, and latency of the proposed method by comparing the said parameters with several distributed estimation algorithms using extensive simulations proposing it for several SG applications. Results prove that the overall performance of the proposed method outperforms other fusion techniques for all considered networks. Under Smart Grid communication environments, the proposed method guarantees for best performance in all fusion accuracy, complexity and energy consumption. Analytical upper bounds for the variance of the final aggregated value at the sink node for structured networks are also derived by considering all major errors.

## 1. Introduction

### 1.1. Motivation

Smart Grid (SG) is the 21st century electric power grid infrastructure that has been proposed to improve the efficiency, reliability, and safety of the 20th century hierarchical, centrally controlled power generation, transmission, and distribution methods [1,2]. These improvements can be established by introducing two-way electrical and information flows for traditional power grids. Smooth integration of renewable energy sources and modern information and communication technologies are the key milestones in the process of transition from a traditional power grid to SG [3,4].

Smart Grid Architecture basically consists of three layers: the Application layer, power layer, and communication layer. Application layer consists of interoperable advanced applications, whereas the power layer integrates renewable energy sources into power generation system and two way communication into all power generation, transmission and distribution system, and customer premises [3]. This paper directly addresses aspects on Application layer and communication layer.

When considering about the communication layer, information and communication technologies provide a platform to collect this information from different entities in SGs. The most attractive platform is the Internet of Things (IoT) which is a network of connected devices [5,6,7]. In SG environments, the end device of the IoT platform is a smart object that has the sensing, processing and networking capabilities [8]. Typical examples are sensor nodes and Radio Frequency Identification Devices (RFIDs) [9,10]. The accuracy of the information produced by these devices is a key factor for reliable power delivery from generation to end user in SGs. The information generated by these devices is contaminated by measurement noise and quantization noise [11,12]. Thus, it is more reliable to gather information from multiple end devices corresponding to a single physical phenomenon. As these devices are not expensive and small in size, it is feasible to deploy these devices in larger quantities. Usually, these devices are not directly interfaced to the internet backbone. There exists a fewer number of units that are capable to collect all information of these devices and route them to the internet backbone. There are back-bone points, back-haul aggregation points, and access points at the end of Wide Area Networks (WANs), Field Area networks (FANs) and Home Area Networks (HANs), respectively. These aggregating nodes are called as sink nodes. Effective information gathering methods are essential to propagate the information from end devices to sink node.

Sensor nodes need to be energy efficient since the sensor nodes have a limited energy. Information gathering using wired cable is not feasible in SG environments due to electromagnetic interference from power lines, incompatibility to wide area network architecture, and availability only to licensed users despite the available resources of power lines and low latency communication. When the wireless sensors network is equipped with cognitive radio, which dynamically access the spectrum, the spectrum efficiency increases and interferences and congestion decreases, and communication errors can be further reduced in SGs [13]. In particularly, installation of expensive cables and regular maintenance of them are an additional burden for SGs. The most cost effective and easiest approach is to use wireless medium. It can be realized through modern IoT end devices that have wireless capabilities [14]. However, drastic environmental conditions such as highly caustic or corrosive environments, high humidity levels, vibrations, dirt and dust, which exist in SG environments can cause high bit error rates during transmissions. In addition, wireless communication is prone to multipath fading and channel characteristic can be dynamic. Therefore, bit errors which occur during the wireless communication added with quantization and sensor noise will degrade the overall accuracy of the sensed parameter.

Bit errors can be reduced to a certain level by information retransmissions and by using forward error correction methods [15]. The trade-off is excessive resource consumption such as energy and channel capacity. As energies of the sensor nodes are also limited, retransmission must be limited. It is specified in [15] that retransmission of a whole information frame/packet to correct a few error bits in an information frame/packet is a waste which confirms the preceding argument. Furthermore, restrictions have been made to maximum number of retransmission attempts in wireless standards [16,17]. These retransmissions are acknowledgment-based retransmissions. In harsh environmental conditions in SGs, these retransmission frames/packets and acknowledgment frames/packets themselves can be lost.

Additionally, low latency communication requirement for mission critical applications, heterogeneous network structure and traffic, and resource (energy, memory and processing) constraints are challenges that exist for wireless sensor networks in SGs [3,13,18]. Latency is the network delay, which is expressed as a time for a data frame/packet to successfully travel from one point to another. Intelligent cognitive radio-based SG can greatly reduce the latency and are aware of heterogeneous networks achieving the required quality of service for SGs [13,19]. The latency requirement specifies that maximum tolerable latency should be 10 ms when the power grid is not broken down into asynchronous sections (non-islanding) and 100 ms for islanded (asynchronous) smart grid systems [20]. Preceding latency requirement was specified for the whole smart grid considering all the applications. However, the latency requirement can vary vastly based on the specific smart grid application [1]. The general recommendation is to use wired media such as fiber optic cables or transmit wireless using Wi-MAX to maintain latency less than 10 ms in Smart Grids covering a bigger area such as WANs. The performance analysis considered in this context particularly aims to address SGs consisting of FANs and HANs in which there is no requirement for mission critical applications having very low latency requirements, but having applications which have a maximum tolerable latency of 300 ms. Zig-Bee or Bluetooth can be used in HANs and Wideband Code Division Multiple Access (WCDMA) in FANs for Smart Grid WSNs [1]. As the transmission distance is relatively low (<10 km per link) in HANs and FANs, they can satisfy the low latency requirement of 300 ms for considered applications in this context in SG communication even under low data rates (10s to 100s of kbps).

When considering the application layer in traditional power grids, power disturbances and outages occur due to equipment failures, capacity limitations, and natural accidents. These problems can be largely avoided through real time power grid condition monitoring and controlling. In generation side, renewable energy farms located in remote geographical areas must be frequently monitored. Critical parameters of solar farms such as radiation and temperature values, DC voltage and weather conditions are required to be collected in a timely manner. In distribution and transmission side, power quality, overhead, and underground cable conditions, conductor temperature and dynamic thermal rating must be collected. Gathered information will pave the way to take early precautionary measures. Therefore, an accurate, less complex and energy efficient data fusion technique is required which causes less network delay. We prove that the proposed statistical data fusion technique is such a fusion method that targets Specific SG applications such as Home Energy Management (HMI), Advanced Metering Infrastructure (AMI), outage management, demand response management, asset management, distributed energy resource and storage, vehicle to grid energy transferring, electric vehicle charging, etc., in which latency requirement is higher than 300 ms [3]. The security aspect of these SG applications are not considered in this context.

IoT end devices have short range transmission capabilities and energy constraints [8]. Thus, centralized estimation by direct information transmission between IoT end devices and internet back bone connected devices are not feasible [21]. Therefore, multi-hop transmissions are required. The chain-based and tree-based topological structures are more realistic to use for multi-hop transmissions in SG environments. For example, the chain structure is well suited to deploy on overhead/underground cables and thermal conductors, whereas the tree structure is well suited to be deployed in larger areas such as wind or solar farms. Furthermore, these structures are simple to implement and easier to scale. Transmitting all the information collected in end devices is the simplest multi-hop information gathering approach. However, this introduces an unnecessary redundant information transmissions that require excessive resources in terms of energy compared to serial distributed detection [22]. Therefore, effective information fusion technique of distributed estimation is needed to combine the information at intermediate devices [23,24].

### 1.2. Review of Data Aggregation

Conventional information fusion functions, which can be incorporated irrespective of parallel of serial information fusion, are type-sensitive, and type threshold symmetric functions such as Average (AVG), Maximum (MAX), Minimum (MIN), Mode, mean of k largest values, etc. [25]. For single bit quantization of data, for instance in the case of decision fusion; OR, AND, K out of N, MAJORITY functions has been used where the performance of information accuracy depended on the measurement frequency and number of nodes [26,27,28]. The Chair Varnish Rule has been presented as the optimum one-bit fusion technique, but in this technique, it is needed to know the probability of detection and failure for all sensor nodes at the fusion center which is a major drawback [29,30]. These information fusion functions combine the information frames/packets from multiple nodes to a single frame/packet. Thus, redundant data transmission can be minimized. Although it is beneficial in terms of energy consumption, this might reduce the accuracy. For example, a single erroneous data can add a biased to the resultant average value when AVG is employed as the information fusion function. Furthermore, MAX and MIN choose only extreme values in information fusion process and do not combine different information to reduce the noise level. Thus, there is a high possibility to vary the fused information from the true value at intermediate devices. It can lead to huge failures in power delivery and stability of SGs due to the unreliable information.

In centralized/parallel estimation, the fusion estimate is produced at a single fusion center where all the sensor nodes transmit their local observations. The major drawback of this aggregation mechanism is that network lifetime is shorter than decentralized approach, as the distant nodes consuming larger amount of energy for direct transmission to the fusion center [22]. An improved version of AVG known as support degree fusion had been used as one of the data fusion techniques for centralized estimation. In this algorithm, the support degree is the amount by which the measurements by each sensor are close to each other. At the fusion center, measurements from each sensor are weighted by the support degree to obtain the estimate using the power average operator without the awareness of any probability distribution [31,32,33].

Decentralized estimation under distributed detection has been a research area over the last few decades. Linear state estimation fusion techniques such as Maximum Likelihood Estimate, Linear Minimal Variance Estimate, and Best Linear Unbiased Estimate (BLUE) had been invented in 1990s [34,35,36,37,38]. These fusion techniques use local estimates and covariance between local estimates to compute a global estimate. More recent trend is to use Kalman Filtering. During the first decade of the 21st century, consensus-based Kalman filter fusion techniques have been proposed [39,40,41,42]. Consensus refers to the general agreement among the sensors. In the preceding algorithm, sensors exchange local information or state estimates among the neighbors during each observation period to reach a consensus on the global estimate known as Consensus Estimation. In between consensus estimates, information is broadcasted to neighbors and fused where state estimates and error covariances are updated. Therefore, broadcasting with neighbors takes place iteratively in between consensus estimations, this algorithm will consume more energy and is less suitable to SG environments.

Diffusion strategies had been developing since early 2010s, which show faster convergence and lower mean square error than consensus strategies [43]. Diffusion strategies shown in [44,45] include standard Kalman filtering update, diffusion update step, and a time update step. At every time instant, a given node will broadcast local observations for Kalman filter update and intermediate estimate for diffusion update. During the diffusion update step, a diffusion matrix is incorporated which has a direct influence on the performance. One realization of diffusion matrix is by Covariance Intersection (CI) method using the information of error covariance [45,46].

### 1.3. Diffusion Kalman Filtering for Comparison with Proposed Method

In this paper, we analyze the performance of the information fusion method proposed in our previous work [11] under various criteria, further proposing it to be compatible for SGs that can improve the information accuracy and reduce the energy consumption. We incorporate the statistical properties of three major error sources that occur in IoT platform: measurement noise, quantization noise, and transmission noise to the information fusion function. With the proposed statistical information fusion approach, our main intention is to achieve the maximum improvement in information accuracy with a single information transmission in each hop. Therefore, we can save the excessive resource consumptions in conventional approaches that are used to improve the information accuracy Even though similar approach is found in [47], it lacks a performance analysis considering the channel bit errors. As it was analyzed in the motivation section, SGs favor high bit error rates. Therefore, the accuracy of the proposed method under high bit errors must be considered. We evaluate the performance of the proposed method in chain and tree network structures comparing its performance with conventional Average fusion and very recently proposed diffusion Kalman Filtering given in [48]. This mechanism is well suitable for comparison since it also does not exchange raw measurements with neighbors like the proposed method. Work presented in [46,48] has been proved to be simpler and accurate than [45] as the former algorithms do not exchange raw measurements. They are also proven to be energy and bandwidth efficient than exchanging raw measurements. Instead of broadcasting observations, they have an individual update and local update of state. Algorithm 1 presented in [46] has been proved to be more accurate than Estimated Based Diffusion Kalman Filter (EBDKF) in [48], but at the expense of communication and computational resources. EBDKF shows more accuracy and have less communication burden compared to Algorithm 2 given in [46] under low sampling periods. Therefore, we choose EBDKF along with conventional Average method to compare with the proposed method for smart grids.

Improvements in both information accuracy and network lifetime of the proposed statistical approach are shown through extensive simulations in bit level and numerical level. The computational complexity of the proposed statistical approach is also investigated comparing with preceding two fusion functions. We also derive analytical upper bounds for variances of the final fused information.

### 1.4. Organization of the Paper

The rest of the chapter is organized as follows. Section 2 presents the Materials and Methods. In-depth analysis of the statistical data fusion approach in chain and tree routing structures as well as bidirectional graph networks are discussed. In Section 3, both analytical and simulation results are presented. The data accuracy, computational complexity and energy consumption are evaluated. Finally, we conclude this paper in Section 4.

## 2. Materials and Methods

### 2.1. Problem Formulation

Consider an IoT platform with K distributed nodes. These nodes are end devices of the IoT platform. Each node k has a measurement $s_{k}$ of a deterministic parameter θ given by Equation (1):

(1) $$s_{k}=\theta +n_{k}$$

where $n_{k}$ is the measurement noise with zero mean and variance of ${{\sigma^{2}}_{s}}_{k}$. If the node k does not have any child node, it quantizes sk into a discrete value $x_{k}$. Otherwise, it combines all the received data from its child nodes and $s_{k}$ together and obtains the quantized discrete output $x_{k}$. Here, we assume that $s_{k}$ is bounded by [−W;W]. The probabilistic scheme in [49] is used to obtain the $x_{k}$ with quantization resolution $L_{k}$. $x_{k}$ is then transmitted to the sensor *k*th parent node. When the quantized output $x_{k}$ propagates through a wireless channel, the signal is attenuated and contaminated by channel noise. The received signal at the parent node can be given by Equation (2) from our previous work in [11] as

(2) $$r_{k}=\left(1-2p_{k}\right)\left(\theta +n_{k}+w_{k}\right)+\gamma_{k}$$

where $w_{k}$ is the quantization noise and the mean of the quantization noise is equal to zeros and its variance is upper bounded by ${\delta_{k}}^{2}$, which is equivalent to $W^{2}/{\left(2^{L_{k}}-1\right)}^{2}$. $\gamma_{k}$ has zero mean and variance ${\epsilon_{k}}^{2}=\left(4W^{2}\left(2^{L_{k}}+1\right)p_{k}\right)/\left(3\left(2^{L_{k}}-1\right)\right)$. The bit error probability between the node *k* and its parent is denoted by $p_{k}$.

### 2.2. Statistical Information Fusion Function

Let k be a node that fuse information where k=1,2,…,K. The set that consists of all child nodes is denoted by $N_{k}$ with size $e_{k}$. The received information from child nodes are given as $r_{i}$ where $r_{i},i\epsilon N_{k}$. The information fusion at node k can be expressed using Equation (3) as

(3) $${\hat{\theta }}_{k}=f\left(s_{k},r_{1},r_{2},\ldots ,r_{i};\ldots ,r_{e_{k}}\right)$$

where *f*(.) is the information fusion function that combines the received information. In smart grid environments, three major types of errors: transmission errors, quantization errors, and measurement errors usually exist in the received information. Statistical estimation can be applied to the information fusion functions and exploit the power of statistics of error sources to obtain accurate information fusion results.

### 2.3. Accuracy Measurement Metric

Information accuracy is a fundamental requirement in any information fusion approach. Square of the difference between the estimated value at the node *k* and the true value is averaged for the total number of fusion estimates produced by the node *k* [44]. That is, for the estimated value ${\hat{\theta }}_{K}$, true value θ and number of fusion estimates $n_{k}$, the Mean Square Error (MSE) of node k is defined using Equation (4) as

(4) $$MSE_{k}=\left({|{\hat{\theta }}_{k}-\theta |}^{2}\right)/n_{k}$$

Network MSE ($MSE_{network}$) is obtained by averaging $MSE_{k}$ for all nodes in the network. $MSE_{network}$ is a numerical level performance metric that gives an overall insight of the information accuracy. The error sources have different levels of impacts on different bits in an information frame/packet.

MSE is a generic accuracy measurement metric that does not depend on the actual value of the measurement parameter. However, the reliability specifications for smart grid applications intended in this context specify that the Percentage Absolute Relative Error (PARE) of the estimate must be upper bounded by 1% [50]. PARE can be calculated using Equation (5).

(5) $$PARE=(|{\hat{\theta }}_{k}-\theta |*100)/\theta$$

We do not use percentage absolute relative error since it is a relative parameter depending on the value of the estimate such that the error percentage of the results, which are obtained will be depending on the actual value of the measurement as evident from Equation (5). To keep our results generic, we use sink node’s MSE and network MSE instead of absolute percentage error. Therefore, we assume that the all MSE values obtained in this context corresponds to absolute percentage error less than 1% meeting reliability requirements of SG applications targeted in this context.

Average error probability at each bit index is a good performance metric to obtain detail bit level accuracy information at the sink node.

### 2.4. Computational Complexity Measurement Metric

Computational complexity of information fusion function is an important measure that determines the feasibility of the function in practice. Ultralow power microprocessors are commonly used in IoT end nodes and they have low computational capabilities. The eight-bit Almega1281 is one of the popular microprocessors used in many nodes such as IRIS, MicaZ and Mica2. These microprocessors support multiple clock frequencies. The number of CPU cycles to execute the information fusion function is a measurable parameter. Thus, we use the time taken to execute the information fusion function ($T_{A}$) as the performance metric to measure the computational complexity. It is expressed by Equation (6) as

(6) $$T_{A}=\frac{Number\;of\;CPU\;Cycles}{Clock\;Frequency}$$

Note that the impact of $T_{A}$ on energy consumption cannot be simply neglected.

### 2.5. Energy Consumption Model

An analytical model given in [51] is used to measure the total energy consumption in data fusion. This model includes details such as consumed energy by frame/packet retransmissions, acknowledgments, frame/packet length, and transceiver processing units. In addition, we include the energy consumption of microprocessor for data fusion as it is an important parameter in low power devices.

The number of transmission attempts of a node can be given by a random variable Xε{1,2,…,N} where N is the maximum number of transmissions including the first transmission. Similarly, the number of acknowledgments can be denoted with a random variable Yε{1,2,…,N}. The transmitter receives 0 acknowledgment if all transmission or acknowledgment attempts fail. The total energy consumption of the transmitter to deliver a frame/packet is given by Equation (7) as

(7) $$E_{TX}=X\left(P_{c}+P_{t}/\eta \right)\left(L_{d}/R_{d}\right)+Y\left(P_{r}L_{a}/R_{a}\right)$$

$P_{t},P_{r},P_{c},$ and η denote the transmit power, receiving power, circuit power, and efficiency of the power amplifier, respectively. The transmission rate of information and acknowledgment frames/packets are defined by $R_{d}$ and $R_{a}$. Lengths of information and acknowledgment frames/packets are given by $L_{d}$ and $L_{a}$ respectively. Similarly, we can compute the total energy using Equation (8) to receive an information frame/packet as

(8) $$E_{RX}=X\left(P_{r}L_{d}/R_{d}\right)+Y\left(P_{c}+P_{t}/\eta \right)L_{a}/R_{a}$$

The energy consumption for the information fusion is measured by Equation (9) as

(9) $$E_{A}=T_{A}*I_{CPU}*V_{CPU}$$

where $I_{CPU}$ and $V_{CPU}$ denote the current and voltage consumption of the microprocessor respectively. Thus, the total energy consumption of a single link is given by Equation (10) as

(10) $$E_{link}=E_{TX}+E_{RX}+E_{A}$$

As there are K−1 links in a gathering process that consists of K nodes, the total energy consumption of the network can be given by Equation (11) as

(11) $$E_{Total}=\sum_{i=1}^{K-1}E_{link,i}$$

In this paper, we use the network lifetime as the performance metric. It is defined as the maximum number of data collecting cycles in a network until all nodes in the network are alive.

### 2.6. Estimation Based Diffusion Kalman Filtering (EBDKF)

The general multidimensional state space model given in [48] is employed in this context to compare with the proposed method. The model basically consists of four update equations, which are updated in each node of the network at each iteration step. The first estimate is known as the individual estimate at *i*th iteration step ${\hat{x}}_{k,i|i}^{ind}$ given by Equations (12) and (13) as,

(12) $${\left({\hat{p}}_{k,i|i}^{ind}\right)}^{-1}={\left({\hat{p}}_{k,i|i-1}^{ind}\right)}^{-1}+{H_{k,i}}^{T}{R_{k,i}}^{-1}H_{k,i}$$

(13) $${\left({\hat{p}}_{k,i|i}^{ind}\right)}^{-1}{\hat{x}}_{k,i|i}^{ind}={\left({\hat{p}}_{k,i|i-1}^{ind}\right)}^{-1}{\hat{x}}_{k,i|i-1}^{ind}+{H_{k,i}}^{T}{R_{k,i}}^{-1}z_{k,i}$$

where $H_{k,i}$ is the individual observation matrix, ${\hat{P}}_{k,i|i}^{ind}$ is a calculated covariance matrix, and $R_{k,i}$ is the sensor noise covariance matrix. These individual estimates are then distributed to neighbors and a local estimate ${\hat{x}}_{k,i|i}^{loc}$ is calculated based on individual estimates received from the neighbors as given in Equations (14) and (15).

(14) $${\left({\hat{p}}_{k,i|i}^{loc}\right)}^{-1}=\sum_{l\epsilon N_{k}}^{}{\left({\hat{p}}_{l,i|i-1}^{ind}\right)}^{-1}$$

(15) $$\left({\hat{x}}_{k,i|i}^{loc}\right)=\left({\hat{p}}_{k,i|i}^{loc}\right)\sum_{l\epsilon N_{k}}^{}{\left({\hat{p}}_{l,i|i-1}^{ind}\right)}^{-1}\left({\hat{x}}_{l,i|i}^{ind}\right)$$

where $\left({\hat{p}}_{k,i|i}^{loc}\right)$ is the local covariance matrix calculated based on received individual covariance from neighbors. $\left({\hat{x}}_{k,i|i}^{loc}\right)$ is the local estimate of the neighbors and sensor node itself individual estimates. These local estimates are broadcasted to neighbors and diffusion update occurs at each of the sensor nodes once the local estimate broadcasted from each of the neighbors are receive as shown in Equation (16).

(16) $$\left({\hat{x}}_{k,i|i}^{dif}\right)=\sum_{l\epsilon N_{k}}^{}c_{l,k}\left({\hat{x}}_{l,i|i-1}^{loc}\right)$$

where $\left({\hat{x}}_{k,i|i}^{dif}\right)$ is the diffusion estimate and $C_{l,k}$ is the diffusion matrix which is determined based on the relative degree rule in Equation (17).

(17) $$c_{l,k}=\frac{n_{l}}{\sum_{s\epsilon N_{k}}^{}n_{s}}$$

The degree of a node is total number of neighbors and node itself. Finally, time update is done based on Equations (18) and (19).

(18) $$\left({\hat{x}}_{k,i+1|i}^{ind}\right)=F_{i}\left({\hat{x}}_{k,i|i}^{dif}\right)$$

(19) $$\left({\hat{p}}_{k,i+1|i}^{ind}\right)=F_{i}\left(\left(b_{k,k}^{-1}\right)\left(p_{k,i|i}^{loc}\right)\right){F_{i}}^{T}+G_{i}\left(\left(b_{k,k}^{-1}\right)\left(Q_{i}\right)\right){G_{i}}^{T}$$

In this time update step, individual estimates to be used in the individual update step of next iteration are calculated. In Equations (18) and (19), $F_{i}$ and $G_{i}$ are coefficient matrices associated with system state and process noise, respectively. $b_{k,k}^{-1}$ is the degree of the *k*th node.

### 2.7. Network Latency

The propagation delay of a link ($t_{pr}$), frame/packet transmission/reception delay of a link ($t_{tr}$), average waiting time in queue per link for one way ($t_{qu}$), fusion time ($t_{fu}$), Maximum Link length out of links connected to neighbor nodes ($l_{i}$), data rate (R), frame/packet size (P), number of Hops (N) of the longest path of the network and Average number of retransmission attempts (K), and career propagation speed (c) are the main determinants of Network Latency of WSNs. Using these parameters, the network latency (TL) for a hierarchical network employing proposed method and AVG can be given as shown in Equation (20).

(20) $$\begin{array}{cc} TL= & \left(\left(2*\left(N-1\right)\left(K+1\right)\right)*\left(t_{pr}+t_{tr}+t_{qu}\right)\right)+\left(\left(N-1\right)*\left(t_{fu}\right)\right)\\ = & \left(N-1\right)*(\left(\left(2\left(K+1\right)\right)*\left(t_{pr}+t_{tr}+t_{qu}\right)\right)+\left(t_{fu}\right))\\ = & \left(N-1\right)*(\left(\left(2\left(K+1\right)\right)*\left(\frac{l_{i}}{c}+\frac{P}{R}+t_{qu}\right)\right)+\left(t_{fu}\right)) \end{array}$$

Network latency is calculated for the longest path in the WSN for AVG and proposed methods. Equation (20) considers both data and acknowledgment frame/packet transmissions. The frame/packet has to travel from hop to hop for networks employing proposed method and AVG. However, for networks employing EBDKF, the frames/packets are only exchanged among neighbors such that the corresponding latency Equation is as shown in Equation (21).

(21) $$\begin{array}{cc} TL= & \left(\left(6*2*\left(K+1\right)\right)*\left(t_{pr}+t_{tr}+t_{qu}\right)\right)+\left(t_{fu}\right)\\ = & \left(\left(6*2*\left(K+1\right)\right)*\left(\frac{l_{i}}{c}+\frac{P}{R}+t_{qu}\right)\right)+\left(t_{fu}\right) \end{array}$$

The network latency is independent of N for diffusion techniques as proved by Equation (21). For bidirectional Ad-Hoc networks the proposed method and average methods will also have to fuse only information from neighbors as described in Section 2.10. Therefore, the network latency of such networks employing AVG method or proposed method can be given as in Equation (22).

(22) $$\begin{array}{cc} {TL}_{chain}= & \left(\left(1*2*\left(K+1\right)\right)*\left(t_{pr}+t_{tr}+t_{qu}\right)\right)+\left(t_{fu}\right)\\ = & \left(\left(1*2*\left(K+1\right)\right)*\left(\frac{l_{i}}{c}+\frac{P}{R}+t_{qu}\right)\right)+\left(t_{fu}\right) \end{array}$$

### 2.8. Information Fusion in Chain-Based IOT Platform using Proposed Method

#### 2.8.1. Background

**Figure 1:**
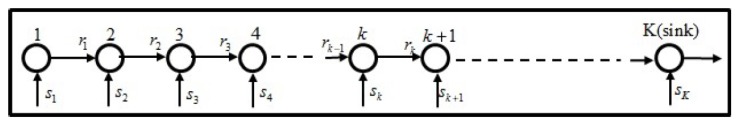
Chain-based network topology.

We first consider a chain-based information fusion technique where all nodes are organized into a linear chain. Each node fuses its own measurement and received information from the nearest neighbor as shown in Figure 1.

The resultant information is then quantized and transmitted to the nearest neighbor in downstream. The information fusion process continues, until the sink node receives the information. We assume that nodes are placed in equal distances. Consider the *k*th node in the chain, it has only one child and that child node is represented by k−1. The linearly scaled received information from the child node is denoted by $r_{k-1}^{{}^{\prime }}$, which is given using Equation (23),

(23) $$r_{k-1}^{{}^{\prime }}=\left(\theta +n_{k-1}+w_{k-1}\right)+\gamma_{k-1}/\left(1-2_{p_{k-1}}\right)$$

Here, $w_{k-1}$ is the quantization noise and the mean of the quantization noise is equal to zeros and its variance is upper bounded by ${\delta^{2}}_{k-1}$, which is equivalent to $W^{2}/{\left(2^{L_{k-1}}-1\right)}^{2}$. $\gamma_{k-1}$ has zero mean and variance $\epsilon_{k-1}^{2}=\left(4W^{2}\left(2^{L_{k-1}}+1\right)p_{k-1}\right)/3(2^{L_{k-1}}-1)$. The *k*th node in the chain fuse $\gamma_{k-1}^{{}^{\prime }}$ and its own measurement $s_{k}$ together. As the mean and the approximated variances of the $\gamma_{k-1}^{{}^{\prime }}$ and $s_{k}$ are available, the weighting property of the BLUE estimation [52] can be used to fuse the data to provide the fusion estimate given by Equation (24) as,

(24) $$\hat{\theta }={\left(1/\sigma_{r_{k-1}^{{}^{\prime }}}^{2}+1/\sigma_{s_{k}}^{2}\right)}^{-1}\left(r_{k-1}^{{}^{\prime }}/\sigma_{r_{k-1}^{{}^{\prime }}}^{2}+s_{k}/\sigma_{s_{k}}^{2}\right)$$

$\sigma_{r_{k-1}^{{}^{\prime }}}^{2}$ is the approximated variance of the $r_{k-1}^{{}^{\prime }}$. The variance of the quantized message at *k*th node is obtained using the inequality Equation (25) as,

(25) $$\sigma_{{\hat{\theta }}_{k}}^{2}\leq \delta_{k}^{2}+{\left(1/\sigma_{r_{k-1}^{{}^{\prime }}}^{2}+1/\sigma_{s_{k}}^{2}\right)}^{-1}$$

Inequality Equation (25) can be further simplified to Equation (26) and the details are given in Appendix A (*k* = 2 scenario).

(26) $$\sigma_{{\hat{\theta }}_{k}}^{2}\leq \delta_{k}^{2}+\left(\sigma_{r_{k-1}^{{}^{\prime }}}^{2}/4\right)+\left(\sigma_{s_{k}}^{2}/4\right)$$

Equation (26) can be further expanded using a similar approach in [53] as given in inequality Equation (27).

(27) $$\begin{array}{cc} \sigma_{{\hat{\theta }}_{k}}^{2} & \leq {(1/4)}^{k-1}\sigma_{s_{1}}^{2}+\sum_{j=2}^{k}{(1/4)}^{k-j+1}\sigma_{s_{j}}^{2}\\ & +\sum_{j=1}^{k}{(1/4)}^{k-j}\delta_{j}^{2}+\sum_{j=1}^{k-1}{(1/4)}^{k-j}\left(\varepsilon_{j}^{2}/{\left(1-2p_{j}\right)}^{2}\right) \end{array}$$

Equation (27) is an analytical upper bound for the variance of the quantized resultant information at any sensor node k in the chain. Substituting k by K, an analytical expression for variance at the sink node can be obtained.

Only the knowledge of the mean and the approximated variances of the $\gamma_{k-1}^{{}^{\prime }}$ and $s_{k}$ is required for this fusion technique. The knowledge on Probability Density Functions (PDFs), which was used to obtain such noise variances, is not required in generating the fusion function. For instance, the output of the fusion function for same mean and variance of $\gamma_{k-1}^{{}^{\prime }}$ and $s_{k}$ which can be generated using any distribution such as Gaussian, Normal, Poisson, Chi-Square, etc. will have the same fusion result. Therefore, the proposed method does not require knowledge on PDF for information fusion.

#### 2.8.2. Method of Performance Evaluation

We conduct a series of simulations using OMNeT++ 5.1 academic edition software platform to evaluate the performance of the proposed statistical information fusion approach comparing the testing parameter with results of AVG fusion and Estimation Based Diffusion Kalman Filter method of fusion. Both fusion accuracy and network life time are considered. OMNeT++ is an open source object-oriented modular discrete event network simulation framework having a generic architecture [54]. In [55], the authors compared the performance of OMNeT++ and Network Simulator 2(NS2), and concluded that OMNeT++ performs better than NS2 for large WSN. As our simulations also contain sensor nodes containing 20 to 240 nodes, we selected OMNeT++ for simulations. As it is impractical to simulate all three algorithms of data fusion at the same time, they were simulated separately in OMNeT++ environment and some final results of three fusion techniques were exported separately to MATLAB environment for graphical analysis, while some were integrated in the OMNeT++ environment appropriately. A stationary, time invariant chain-based network topology is generated by deploying nodes in equal distances. We assume that MICA2 wireless sensor nodes are used in the network as IoT end devices. The BER of each link is determined based on the analytical model given in [56]. We set the transmission power $P_{t}=0$ dBm, reference distance $d_{0}=1$ m, power loss at reference distance $PL(d_{0})=55$ dBm, the noise floor P(n)=−115 dBm and the parameters for path loss exponent (n), and shadowing effects (σ) in Equation (29)) are set to 3.3 and 4. The modulation scheme selected for wireless communication was Non-Coherent Frequency Shift Keying so that $BER(P_{e})$ is given by Equation (28) [56].

(28) $$P_{e}=\frac{exp^{\frac{-\gamma (d)}{2}}}{2}$$

where γ(d) is the signal to noise ratio at distance d from the source which is given in dB by Equation (29) as,

(29) $$\gamma \left(d\right)indB=P_{t}-\left(PL\left(d_{0}\right)-10*n*log_{10}\frac{d}{d_{0}}+X_{\sigma }\right)-P_{n}$$

All other parameters are as specified in [56]. Unless otherwise specified, the $L_{k}$ is set to 8. Heterogeneous measurement noise variances are considered and they are generated for all fusion techniques as given in Equation (30) subjected to an upper bound for fair comparison of three different fusion techniques.

(30) $$\sigma_{s_{k}}^{2}=R_{k,i}=b_{0}+{a_{0}}^{2}\chi^{2}\left(1\right)$$

Here, $\chi^{2}\left(1\right)$ is the chi squared distribution with degree of freedom 1, $b_{0}$ represents the network wide noise variance threshold, and $a_{0}$ gives the underlying variation from the nominal minimum. Noise variance is calculated in this manner to create heterogeneous sensing conditions between sensors of the same network and to create heterogeneous network conditions among initializations. This variance is calculated at the initialization of the network for each node and will be a constant there after. Gaussian distribution is used to generate instantaneous noise ($n_{k}$) of a particular sensor node. This noise is added to the actual parameter to get the sensor measurement as shown in Equation (1). The Gaussian distribution’s mean is set to zero and variance is set to the value generated at the network initialization using Equation (30) for a particular sensor node. Note that different sensor nodes of the same network will have different noise variances as they are generated from Chi-Square distribution as it was shown in Equation (30). For a particular sensor of a given network, the variance generated using Equation (30) is a constant. That means each time a particular sensor node takes a measurement; the noise is generated using the same Gaussian distribution. However, when a network is initialized again, Equation (30) will make sure that a particular sensor will get a different value for variance making the PDF of sensor measurements different. Noise variance is upper bounded to 0.7 and sensor measurements are lower and upper bounded by −10 and 10 respectively. As measurements are bounded by [−10,10], we bound the quantized value of a fused output by previous range. Any fused result higher than 10 is set to 10 and a fused result lesser than −10 is set to −10. We assume that the network measures a deterministic parameter θ, which is equal to 1.8 and bounded by [−10,10] (i.e., $W^{2}=100)$. This upper and lower bounding is very practical. Consider a sensor network measuring the frequency of a transmission line having an expected value of 50 Hz. Here, the measurements can be lower and upper bounded by 45 Hz and 55 Hz, respectively, similar to what we have done in these experiments which is very practical.

For fair comparison of the proposed method with EBDKF in [48] we consider the general multi-dimensional state-space model given in [44,48] as shown in Equations (31) and (32).

(31) $$x_{i}=F_{i-1}x_{i-1}+G_{i-1}w_{i-1}$$

(32) $$z_{k,i}=H_{k,i}x_{i}+v_{k,i}$$

As only one parameter is estimated in our context, the coefficient matrices *H*, *G*, and *F* in Equations (31) and (32) reduce to one dimension. Even though the process noise (Q) is set to 4 in [44], we select the state noise ($w_{i-1}$) as zero which will result zero process noise variance ($Q_{i}$). This is because the addition of a process noise even worsens the BER performance of EBDKF for update equations in [48]. In particular, we chose the best-case performance wise scenario for EBDKF to compare with the proposed method which exists when there is no process noise. Then, it will be crystal clear that if a data fusion method outperforms the accuracy of EBDKF under its best performance scenario, then the considered fusion method will outperform EBDKF under any other scenario. Therefore, the value of G is not relevant for zero process noise scenario as it can be observed from Equation (31); but we set it to zero. We set H=1 because observations are not scaled by a factor according to the way that we have modeled sensor observations for proposed method and Average as it was shown in Equation (1). In order to have a fair comparison with AVG and proposed method, the sensor observations of EBDKF are modeled in the same manner. That is the true parameter added with Gaussian noise. Therefore, H=1 for the state-space model. The sensor measurement noise variances ($R_{k,i}$) are generated as given in Equation (30). ${\hat{x}}_{k,0|-1}^{ind}$ is modeled as a continuous uniform random variable in range [−10:10], ${\hat{P}}_{k,0|-1}^{ind}=n_{k}$, where $n_{k}$ is the degree of the *k*th node. We first evaluate the accuracy of estimating a slowly varying(deterministic) parameter for the considered time interval and then later consider the accuracy of a dynamic parameter using the three different fusion algorithms.

### 2.9. Information Fusion in Tree-Based IOT Platform Using Proposed Method

#### 2.9.1. Background

**Figure 2:**
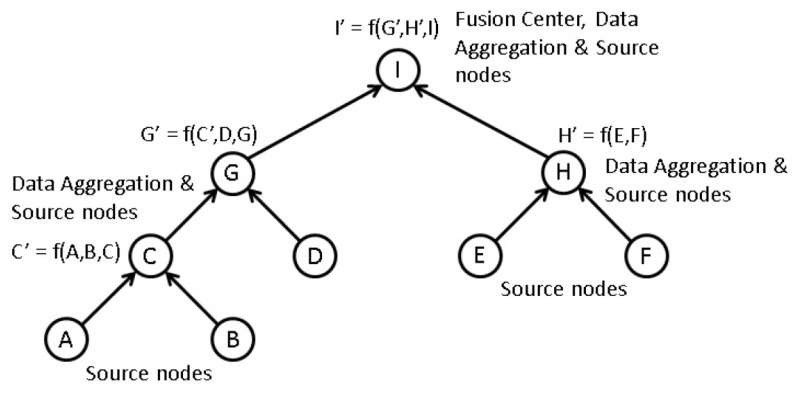
Binary tree-based network topology using data gathering with information fusion function f(..).

Tree-based information fusion is the most generalized hierarchical information fusion scheme. Figure 2 shows a typical binary tree-based hierarchical network.

Although the given network looks simply, it is scalable with respect to the number of nodes. Here, node I represents the sink node which is the root node of the tree. All the other nodes can be either a source node or information fusion node. Nodes A, B, D, E, and F are leaf nodes of the tree that only transmit local measurements to parent nodes in Average and proposed methods of information fusion algorithms acting only as source nodes. The intermediate nodes C, G, and H fuse incoming data from its child nodes with their own local measurements and forward the results to their parent nodes. Therefore, these nodes work as source and information fusion nodes. Any parent node can have multiple child nodes. Figure 2 is a simplified version intended for visual clarification purposes. This kind of network connections can be seen towards the sink node I. Here, the information fusion directions are represented by arrows. In contrast in EBDKF fusion technique, all the nodes aggregate data and sensor measurements due to inherent two-way communication present in diffusion techniques. Therefore, for this technique there is no identifiable root node or leaf nodes unlike in Average or proposed method fusion techniques.

Consider an information fusion node k in the tree that has $e_{k}$ number of child nodes. The weighting property of BLUE estimation [52] can then be applied to obtain the fused data at node k depicted by Equation (33) as

(33) $${\hat{\theta }}_{k}={\left(\sum_{i\varepsilon N_{k}}\left(1/\sigma_{r_{k-1}^{{}^{\prime }}}^{2}\right)+1/\sigma_{s_{k}}^{2}\right)}^{-1}\left(\sum_{i\varepsilon N_{j}}r_{k-1}^{{}^{\prime }}/\sigma_{r_{k-1}^{{}^{\prime }}}^{2}+s_{k}/\sigma_{s_{k}}^{2}\right)$$

variance of the quantized fused information at node k can be shown using inequality Equation (34) as

(34) $$\sigma_{{\hat{\theta }}_{k}}^{2}\leq \delta_{k}^{2}+({\sum_{i\varepsilon N_{k}}\left(1/\sigma_{r_{k-1}^{{}^{\prime }}}^{2}\right)+1/\sigma_{s_{k}}^{2})}^{-1}$$

We can further approximate Equation (34) using a similar approach in [53] to obtain the inequality in Equation (35) as

(35) $$\sigma_{{\hat{\theta }}_{K}}^{2}\leq \delta_{K}^{2}+1/{\left(1+\epsilon_{K}\right)}^{2}\sum_{i\varepsilon N_{K}}\sigma_{r_{k-1}^{⊣}}^{2}+\sigma_{s_{K}}^{2}/{\left(1+\epsilon_{K}\right)}^{2}$$

The proof is given in Appendix A. We can analytically compute an upper bound for the variance of the fused information at the sink node by considering the three major error sources as shown in inequality Equation (36).

(36) $$\sigma_{k}^{2}\leq \sum_{k=1}^{K}(\alpha_{k}\delta_{k}^{2})+\beta$$

Here, we assume $\alpha_{K}=1$ where K is the sink node. We determine $\alpha_{i}=\alpha_{k}\left(1/{\left(1+e_{k}\right)}^{2}\right)$ recursively for all 1≤k≤K and β is computed as given in Equation (37).

(37) $$\beta =\sum_{k=1}^{K}\left(\alpha_{k}\left(1/{\left(1+e_{k}\right)}^{2}\right)\right)(\sigma_{s_{k}}^{2}+\epsilon^{2}/{\left(1-2p_{k}\right)}^{2})$$

Here $i\varepsilon N_{k}$.

#### 2.9.2. Method of Performance Evaluation

The proposed statistical information fusion approach is tested on a tree network using OMNeT++ simulations. Simulation results were exported to MATLAB, as in the case of chain topology for graphical analysis. In these simulations, we analyze the improvements in the data accuracy and the network life of the proposed method compared to AVG and EBDKF data fusion methods. We consider a 300 m × 300 m square region where sensor nodes are randomly deployed. Here, we use a protocol in which the tree-based WSN is built by using the distance between the neighbors as the cost function to ultimately join to the fusion center. That is a given node will connect to another node which has the least distance with the given node. This protocol will maximize the inter-node communication than Minimum Spanning Tree (MST) protocol which minimize the total distance between a given node and the fusion center rather than minimizing inter-node distance. Therefore, this protocol will be more reliable even though the total cost of transmission might be expensive than the MST protocol. This protocol will enhance more child nodes to be connected to an aggregating node than the MST protocol where the average number of child nodes per aggregating node is one. As our intention is to study the accuracy, complexity, and network lifetime when aggregating nodes have more children, i.e., effect of parallel data aggregation along with serial data aggregation, we use the previously said minimum neighbor distance tree without using MST protocol. Therefore this network structure is a more practical and generalized structure due to random placement of sensor nodes and having different inter-node distances than the previously discussed chain network. Here, the Euclidean distance among sensor node is used as the cost function. The sink node is placed at the (150, 150) coordinate which is the center of the square region. Unless otherwise specified, all the other parameters of the network are similar to the parameters specified in the chain-based network simulations except the link length and BER. In chain-based network, we assumed that the nodes are placed in equal distances so that the BER s of all the wireless links are the same. However, in this tree structure resembling a more practical sensor network used in SG environment, since a minimum neighbor distance connection method was used, the link distance varies between different node pairs making the BER of links different according to Equation (29).

### 2.10. Information Fusion in Randomly Placed Non-Hierarchical Bidirectional Graph Based IOT Platform Using Proposed Method

#### 2.10.1. Background

**Figure 3:**
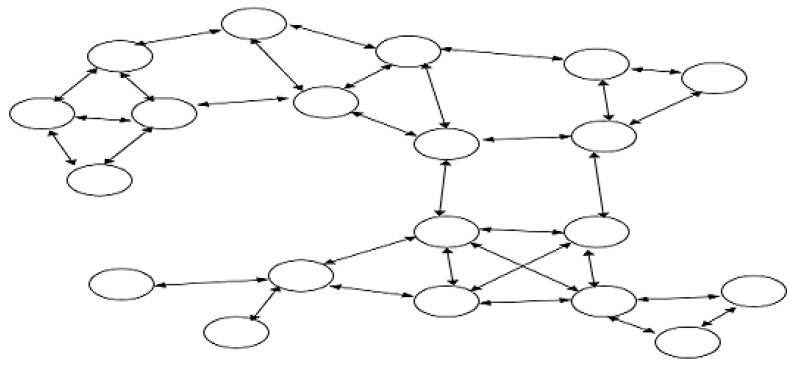
Non-hierarchical Bidirectional network with 20 sensor Nodes.

In a non-hierarchical sensor network, there is no fusion center due to inherent bidirectional communication. In the preceding sections, we analyzed the performance of chain and tree networks for proposed and average fusion which are hierarchical in structure for one directional communication. We also applied the diffusion technique for chain and tree structures using bidirectional communication which converts the unidirectional hierarchical structure into a graph without a fusion center. Diffusion techniques have been originally proposed and tested for ad hoc-type networks which do not have any fusion center in which the sensor nodes are not aware of the network structure as seen in [43,44,45,46,48]. In those networks, a given sensor node is aware of the sensor nodes connected to it known as neighbors. In previous sections, we compared unidirectional average and proposed fusion techniques with the bidirectional diffusion technique for chain structures. In this section, we implement proposed method and average method fusion techniques as bidirectional in order to be used in an unstructured graph, in which the sensor nodes are randomly deployed and are able to communicate with limited number of neighbors due to transmission capability. We employ the network given in Figure 3.

#### 2.10.2. Method of Performance Evaluation

The proposed statistical information fusion approach is tested on a Bidirectional graph using constructed using OMNeT++ simulations. Here, the distance between the sensor nodes are fixed; however, there is no protocol used in defining which sensor nodes will be neighbor node of a particular node as seen in Figure 3. The network constructed can be considered as an instance of an Ad-Hoc network.

The information accuracy of the unstructured network in Figure 3 is tested by comparing the network MSE for three different data fusion techniques. There is no modification to EBDKF algorithm for unstructured graph. But, a modified approach for the proposed method and average data fusion are employed here.

A data cycle of proposed method will consist of obtaining a sensor measurement $\left(s_{k}\right)$ at *i*th data gathering cycle and broadcasting it to neighbors. Then, the received information broadcasted in this manner is fused according to Equation (33). $s_{k}$ in this equation is the sensor’s own measurement at the *i*th data gathering cycle. This broadcasting will be done only one time since the diffusion technique also produce one diffusion estimate per data gathering cycle. In contrast, in consensus approaches, the measurements are broadcasted n times per data gathering cycle until all the nodes reach global estimate of mean of all sensor measurements [39,40,41,42,57]. Similar approach to proposed method is used for the average fusion technique in which only the data fusion technique is different.

## 3. Results and Discussion

### 3.1. Simulations for Chain-Based Network

#### 3.1.1. Simulations for Data Accuracy of a Deterministic Constant (Slowly Varying) Parameter Having Bounded Sensor Noise and Measurements

**Figure 4:**
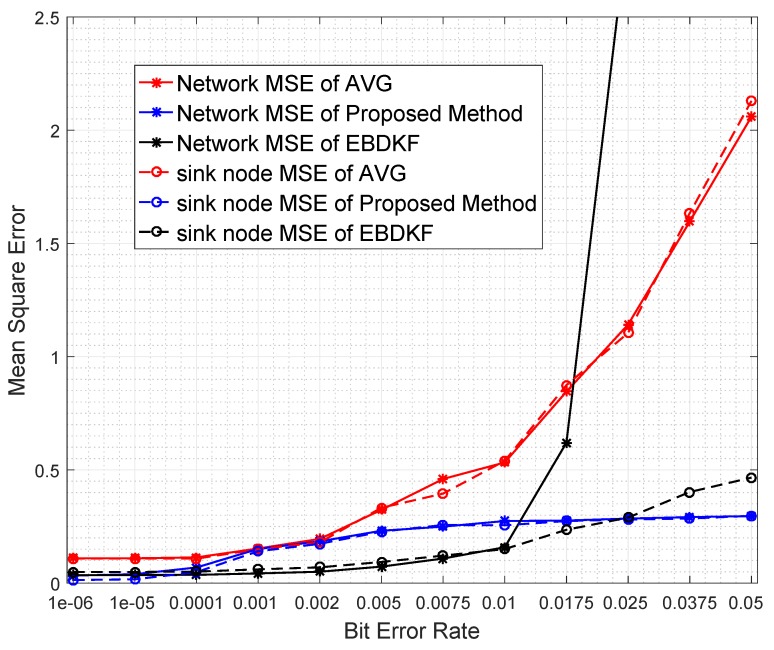
Performance comparison among the proposed statistical, EBDKF, and AVG information fusion methods in a chain-based IoT platform for different BER.

In this section, we compare the $MSE_{network}$ and the $MSE_{sink\;node}$ when the proposed statistical information fusion method; the EBDKF and AVG method are employed in the presence of measurement errors, quantization errors, and transmission errors. We run 600 iterations for a single run in order to get enough steady state measurements while there are multiple number of runs for a simulation. This algorithm is used to get ${\hat{x}}_{k,i|i}^{diff}$ and then as seen in Section 3 in [44]; it is used to obtain the estimate of deterministic parameter θ, having an expectation of 1.8. One of the reasons for selecting 1.8 as the system parameter for analysis is since it is close to zero such that sensor measurements will rarely exceed sensor measurement limit. Also, it is not one of the quantized output values in the implemented quantized scheme of 8 bits as the nearest quantized output values to it are 1.79688 and 1.875. Therefore, even the highest accurate estimation of 1.80 will cause a quantization error of 0.00312 so that effect of quantization error will be reflected on the generated results. In these simulations, we generate different network conditions for different runs and in each network condition, data is gathered 600 times. The final results are obtained by averaging these simulation results. We first analyze the impact of transmission errors. A network that consists of 50 nodes is considered and the BER of a link is increased by reducing the distances between nodes. These values are varied between $10^{-6}$ and 0.05 by changing the distance between two consecutive nodes from 24.45 m to 41.5 m. We generate heterogeneous measurement noise variances by setting $b_{0}=0.3$ and $a_{0}=0.1$ with variance upper bounded by 0.7. Figure 4 compares the Network MSE and MSE at sink nodes of the proposed statistical method, EBDKF, and AVG method.

We can observe that the proposed statistical method outperforms AVG method for the considered BER range. The performance gap increases significantly with the BER. The proposed method also outperforms the EBDKF when the BER, due to communication errors, is greater than 0.015. For very low BER values (<10${}^{-5}$), the accuracy of proposed method is slightly higher than Diffusion Kalman Filtering. It can be observed from Figure 4 that for higher BER (>0.01), the EBDKF algorithm’s network mean square error increases with a rising gradient. That is due to the fact that EBDKF heavily relies on multiple transmissions among neighbors for a single diffusion estimate at an iteration step which can get highly inaccurate under transmission errors. Due to that, as it is evident from Figure 4 EBDKF is superior than the proposed method in terms of information accuracy for moderate bit error rates in the range of $10^{-5}$ to 0.0115. However, EBDKF shows large MSE under high communication errors which can occur in a Smart Grid environment. EBDKF’s accuracy degrades even below average fusion method when BER is greater than 0.0175. However, on the other hand, the proposed method is aware of the communication errors and adjust the weighting coefficient automatically which is $\sigma_{r_{k-1}^{{}^{\prime }}}^{2}$ in Equation (24). When communication errors increase, $\sigma_{r_{k-1}^{{}^{\prime }}}^{2}$ also increases, so that it having a smaller weight in the fusing equation. Due to this as evident from Figure 4, MSE in proposed method increase with a very low gradient with BER.

As the proposed statistical method accurately models the received information, we are able to eliminate any bias that can occur in the final result by dividing each received data with corresponding $\left(1-2p_{k-1}\right)$ coefficient. Furthermore, the use of the weighting property in the conventional BLUE estimator helps to achieve the minimum variance for the received information. Thus, the proposed statistical method is able to maintain the network mean square error less than 0.29 for the considered BER range.

Diffusion techniques do not differentiate between the parent and child nodes; they identify both child nodes and the parent as neighbors. For chain structure employing diffusion techniques, the number of neighbors of the intermediate nodes is always equal to two, whereas the number of neighbors of the first and sink node is equal to one. The number of child nodes per network and the sink node for a WSN employing the proposed statistical method and the AVG fusion techniques are always equal to one. Therefore, the accuracy of the information fusion for network and the sink node will be similar for networks employing AVG and the proposed method, as is evident from the results obtained in Figure 4. When the number of nodes is high, so is the number of intermediate nodes. Therefore, the average number of neighbors for a network employing EBDKF is equivalent to two. But, the sink node will definitely have only one neighbor. The performance gap of EBDKF for network MSE error and MSE of sink node observed in Figure 4 must be due to this difference in number of neighbors of the sink node and the network. For low BER values, the network MSE has been lower than sink node’s MSE. That is because an estimate produced by a diffusion technique becomes more accurate when the number of neighbors is higher under low communication errors. However, the higher number of neighbors will greatly reduce the accuracy under high communication errors for diffusion techniques. That is why the sink having only one neighbor outperforms its network having two neighbors for high BER values. However, still, we can observe that the proposed method’s sink node’s MSE is lesser than MSE of sink node employing EBDKF for BERs higher than 0.025. However, the accuracy of the sink node of EBDKF fusion technique is always lesser than accuracy of a sink node with Average data fusion for all BER s.

**Figure 5:**
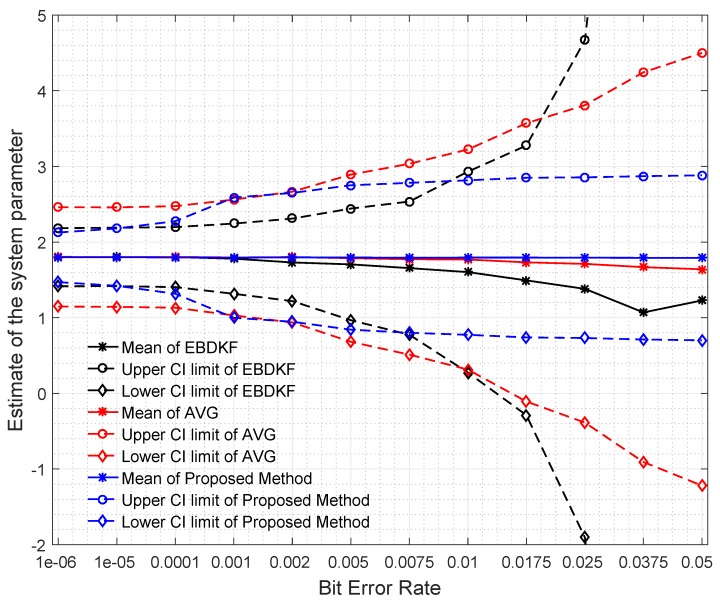
Network Mean estimate and 95% confidence interval limits for a true state of 1.80 compared among the proposed statistical, EBDKF and AVG information fusion methods in a chain-based IoT platform for different BER.

To have a very good understanding about the network’s estimate of the system parameter under different BER for different information fusion schemes, we plot the mean value of the estimate at each node and the mean value of each node’s 95% confidence interval limits as illustrated in Figure 5.

When analyzing the results obtained in Figure 5, it can be seen that for low BER values, the network mean estimate is very close to the system state of 1.8 for all fusion techniques. However, the performance in terms of accuracy is different due to difference in the variance of the estimates of different fusion techniques. This variance can be identified by looking at the size of the 95% confidence interval in Figure 5. In very low BER conditions (<10${}^{-5}$), the 95% confidence interval is also narrow and its width is in decreasing order for Average, EBDKF, and proposed method proving the previous result obtained for network MSE for very low probability of communication errors. This is because we can say that when the confidence interval for the results is higher, so is the network mean square error. The mean estimate of the proposed method decreases slightly and remains very close to the real value of 1.8 when BER increases outperforming the accuracy of mean estimate of other two fusion techniques. However, the mean value tends to deviate more from real value of 1.8 when BER increases for other two data fusion techniques where the AVG method having a moderate deviation and EBDKF having the highest deviation. The reason for observing high network MSE for high BER for average and EBDKF can be explained by looking at the increasing size of the 95% confidence interval for those two methods as evident from Figure 5. In contrast, the proposed method’s confidence interval is only slightly increased with increasing BER, so that it is showing least network MSE for high BER. Further, the average method’s accuracy approaches that of proposed method when 0.001<BER<0.002 as seen from the confidence interval and observable in Figure 4 as well.

**Figure 6:**
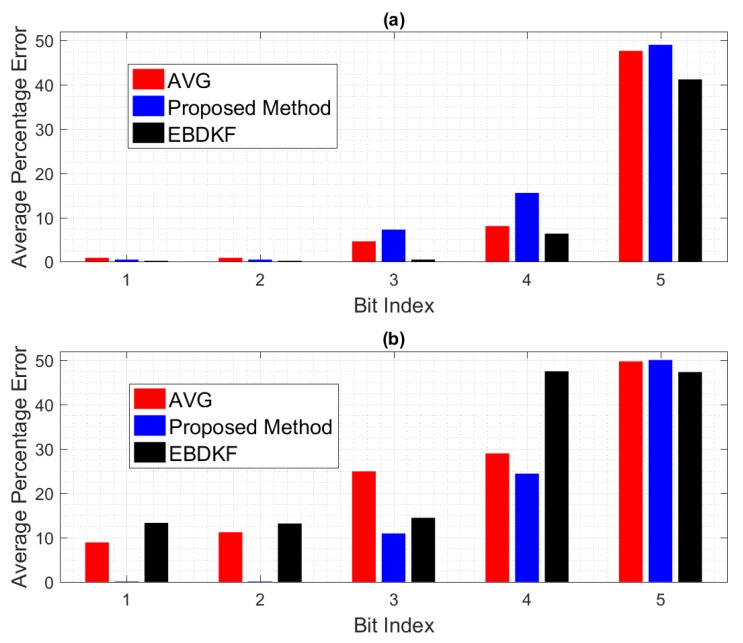
Bit level error performance comparison among the proposed statistical, EBDKF, and AVG information fusion methods in a chain-based IoT platform for (**a**) BER of 0.005 and (**b**) BER of 0.05.

We further analyze the impact of the transmission errors on each individual bit in a data frame/packet. Figure 6 depicts how each individual bit in a data frame/packet experience two different BER conditions. Here, we compare the mean value of bit wise accuracy of the quantized output of fused result of each node with the quantized value of the system parameter. We set 100% error if a bit is altered and 0% error if the particular bit of the quantized fused output is same as the corresponding bit of the quantized value of system parameter. Here, we get the mean of the results for particular bit considering all the nodes to obtain the network bit level performance for first five most significant bits.

The significant bits can drastically change the final value from the true value. For example, the impact of MSB is higher than the total combination of next four significant bits. When BER is 0.005, the average error probabilities of first five significant bits of EBDKF is lesser than both AVG and proposed method. The percentage error of first two Significant bits for proposed and AVG methods remain at very low values but higher than EBDKF. The decreasing order of magnitude of percentage error in first two most significant bits are in the order of AVG, proposed method, and EBDKF. Therefore, this proves why it is seen a high network MSE and high width of 95% confidence interval in Figure 4 and Figure 5, respectively, in decreasing order as AVG, proposed method and EBDKF when BER is 0.005. However, when BER is 0.05, the preceding order changes to EBDKF, AVG, and the proposed method proving that proposed method shows lower error probabilities for the first two most significant bits compared to AVG and EBDKF as seen in Figure 6. When observing the size of the confidence intervals at a BER of 0.05, only the proposed method’s interval is small as seen in Figure 5. The size of the confidence interval is moderately large for Average and very large for EBDKF. Same order is observed for first two MSB s for BER of 0.05 in Figure 6. Therefore, we can use the proposed method to protect these most significant bits successfully under high communication error environments. This explains why we observe a significant performance gap among the three different fusion schemes at BER 0.05 in Figure 4 and Figure 5. It can also be observed from Figure 6 that the percentage error of both third and fourth significant bits of all considered fusion schemes have increased when BER is 0.05 compared to BER of 0.005. However, the percentage error of first two significant bits for proposed method is reduced by a very small amount and that for Average and EBDKF have been significantly increased as the BER is increased.

**Figure 7:**
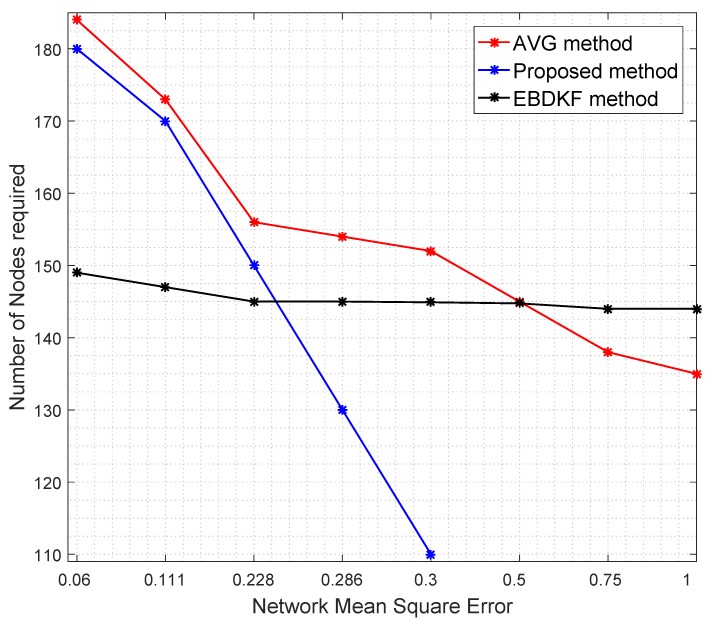
The number of nodes required to achieve a given percentage error using the proposed statistical method, average, and EBDKF data fusion for IoT platform.

Redundant node deployment can be used to improve the accuracy of fusion schemes. We simulate the information fusion process in chain-based network by varying the number of nodes. Here, the distance between the sink node and the furthest node is kept constant at 2500 m. Figure 7 shows the number of nodes required for AVG, EBDKF, and proposed statistical methods to maintain a given network mean square error.

It is evident from Figure 7 that when more sensor nodes are added, the network MSE of all the aggregation schemes decrease. However, the rate of decrement of MSE depends on the aggregation method. To achieve a very low MSE, for instance consider 0.06, as it is evident from Figure 7, we need about 35 additional nodes for AVG and 31 additional nodes for the proposed method than EBDKF. However, to achieve MSE of 0.228, the proposed method will require 10 additional nodes while AVG will require around 21 more additional nodes than required by EBDKF method. When the MSE requirement goes above 0.25, the proposed method outperforms both AVG and EBDKF. For instance, to achieve MSE of 0.30, EBDKF will require 35 more nodes and AVG will require 42 more nodes than the proposed method. These results verify the network MSE obtained in Figure 4. Therefore, using less resources (nodes), the proposed method can achieve an MSE as low as 0.3 than other data fusion techniques considered.

**Figure 8:**
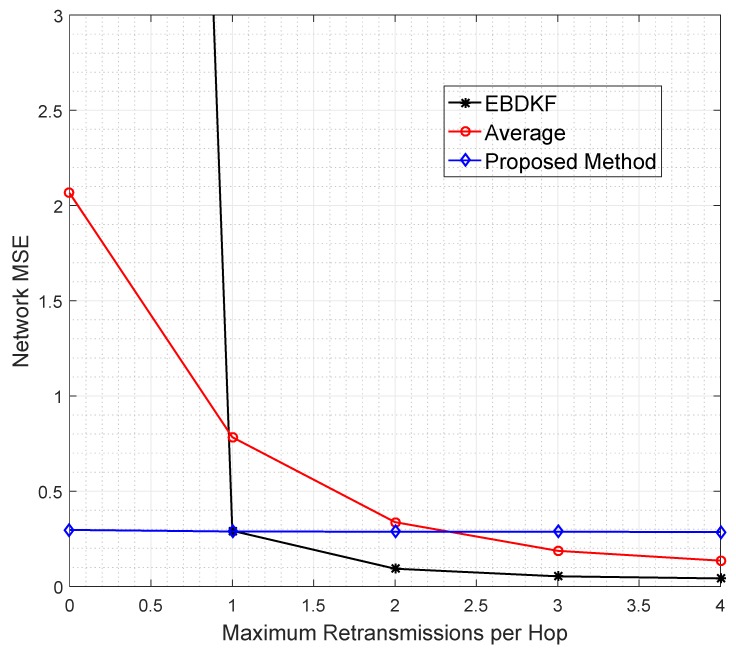
The performance comparison among the proposed statistical, AVG, and EBDKF information fusion methods in a retransmission allowed chain IoT platform.

The data retransmission is another technique used to mitigate the impacts of transmission errors. In particular, we analyze the network MSE vs. maximum number of additional retransmissions allowed per hop per data frame/packet required for AVG, EBDKF, and the proposed statistical method, as shown in Figure 8. Here, we consider an equally spaced 50 nodes in a chain-based network. The BER of a link is equal to 0.05. Here we have made the assumption that communication channel errors are not affecting on acknowledgment frames/packets so that an acknowledgment sent is always received. We vary the maximum number of retransmission attempts for each hop per data frame/packet between 0 and 4.

It is observed that the proposed method maintains the network MSE less than 0.3 under all conditions having a very low gradient suggesting that its network MSE is very slightly decreased in a retransmission allowed network. The data retransmission does not provide any additional benefit for the proposed method to improve the accuracy. On the other hand, for AVG method, it is required to set the maximum retransmission attempts to 3 to reduce the MSE less than 0.30. Further, the EBDKF method shows a very high network MSE when no retransmissions are allowed having least accuracy out of the three fusion methods. But it can improve drastically its network MSE to 0.29 getting almost equal MSE performance to the proposed method and outperforming Average fusion when a maximum of one retransmission is allowed as evident from Figure 8.

Even though retransmissions can reduce network MSE, excessive energy for both information and acknowledgment retransmissions, representing excessive use of communication resources, are the major drawbacks. Therefore, it is evident that the proposed method can outperform both AVG and EBDKF at high BER value like 0.05, as it can achieve least network MSE with least usage of energy and communication resources.

The usage of communication resource for frame/packet transmission in three schemes for chain-based network can be generalized as follows. Assume that there are *k* number of nodes and n is the number of information frames/packets that are transmitted within a single link per data gathering cycle in a chain-based WSN in which retransmissions are not allowed. Also, let *L* be the maximum number of retransmissions allowed for a node per information frame/packet. Depending on the channel conditions, the maximum retransmission attempts might not be used in each link. The number of retransmissions will depend on the BER($P_{e}$). All nodes will transmit at least one time. Then, when L=1, the number of nodes retransmitting one time will directly depend on the value of $P_{e}$. When L=2, it can be assumed to be proportional to ${P_{e}}^{2}$ and so on and so forth. Therefore, total Transmissions (*N*) considering only information frame/packet (without acknowledgments) transfer will be as shown in Equation (38),

(38) $$N=\left(n\right)*\left(k-1\right)*\left(1+\lambda_{1}P_{e}+{\lambda_{2}P_{e}}^{2}+\ldots \ldots +{\lambda_{L-1}P_{e}}^{L-1}+\lambda_{L}{P_{e}}^{L}\right)$$

where $\lambda_{k}$ is a constant. Using Equation (38), an upper bound on number of total transmissions can be derived as depicted in Equation (39) for the cases when $\lambda_{k}{P_{e}}^{k}=1$.

(39) $$\begin{array}{cc} N= & \left(n)*(k-1)*(1+1+1\ldots +1+1\right)\\ = & \left(n)*(k-1)*(L+1\right) \end{array}$$

As proved in the preceding section, L=0,1,3 in the proposed method, EBDKF, and Average fusion methods, respectively, to achieve a network MSE less than 0.3. It is evident that n=1 in proposed method and average method as a node will send only one fused data frame/packet in a link. As there are two $\hat{x_{ind}}$, $\hat{\left(x_{ind}\right)}*\left({(p_{ind}^{curr})}^{-1}\right)$, $x_{loc}$ for each link in EBDKF per data gathering cycle, n=6. Therefore, by substituting in Equation (39) for upper bound, $N_{proposed}=\left(k-1\right)$, $N_{AVG}=\left(4\right)*\left(K-1\right)$, $N_{EBDKF}=\left(12\right)*\left(K-1\right)$. Therefore, when the network MSE is less than 0.3, total retransmissions will be lesser than the upper bound. In order to obtain only retransmission upper bound, L+1 in Equation (38) will have to be replaced by *L*. When acknowledgment frames/packets are also considered, the total transmissions will be even more than the values calculated in preceding sections.

**Figure 9:**
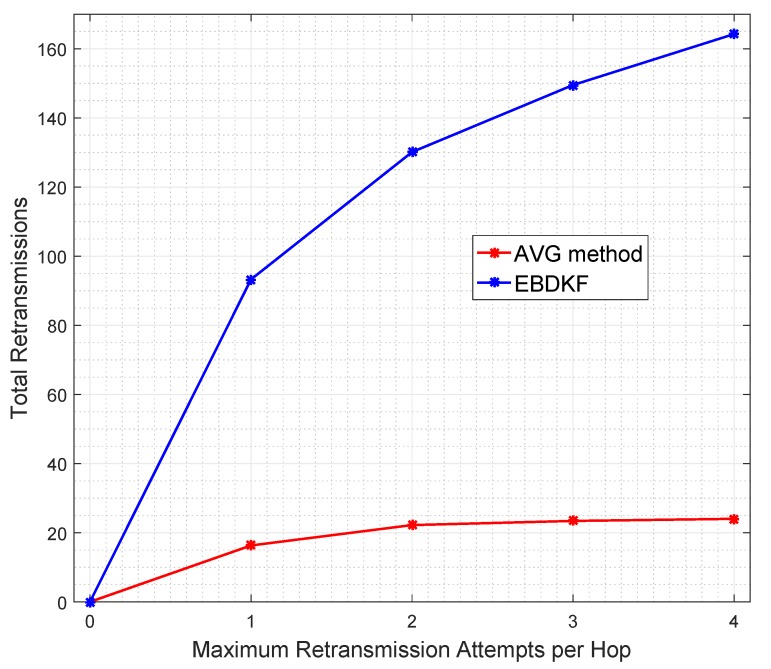
The total retransmission attempts used by AVG and EBDKF fusion methods in a retransmission allowed chain IoT platform.

Although, the information retransmissions have been shown as a solution to improve the accuracy of the information fusion with AVG and EBDKF methods as it was stated in preceding section, they consume additional energy which is a major disadvantage in WSN s. To get the exact number of total retransmissions when BER is 0.05, Figure 9 was obtained to visualize the total retransmission attempts for data frames/packets utilized in the network when the maximum retransmission attempts are limited to one, two, three, and four among EBDKF and AVERAGE fusion techniques. We do not observe the total retransmissions of proposed method, as we came to conclusion that frame/packet retransmissions very slightly improve the accuracy of the estimate generated by proposed method.

It can be clearly observed from Figure 9 that all values are within the upper bound for retransmission of 157 and 294 for AVG and EBDKF when L=3 and L=1, respectively. As observed in Figure 9, approximately total of 24 retransmission attempts are used in AVG method and 93 in EBDKF to achieve a network MSE less than 0.3. These additional retransmissions consume excessive energy for information retransmissions. Therefore proposed method can effectively save transmission energy compared to other fusion algorithms.

#### 3.1.2. Simulations for Data Accuracy of a Deterministic Dynamic Parameter Having Bounded Sensor Noise

**Figure 10:**
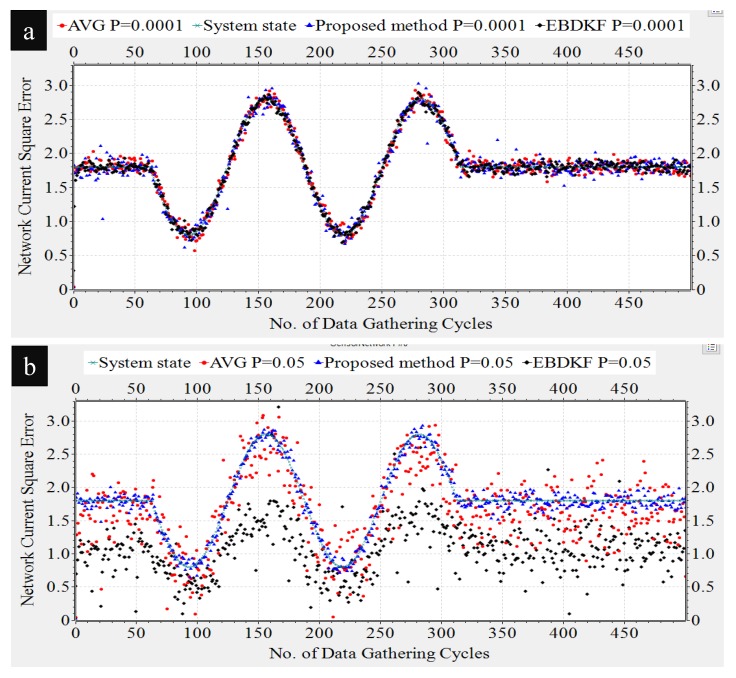
The performance comparison among the proposed statistical, AVG and EBDKF for a dynamic system parameter having communication link with (**a**) BER of 0.0001 and (**b**) BER of 0.05.

The actual value of the system parameter measured by sensor nodes is not a constant value all the time; it can vary over time. For example, when sensor nodes are used to monitor the transmission lines in a smart grid environment [3,4], voltage or frequency fluctuations can occur. Therefore, it is necessary to evaluate the performance of the proposed method for dynamic states to check whether the estimates can track the varying parameter. Here, we simulate the system parameter having a mean of 1.80 and having intermittent sinusoidal fluctuation representing the dynamic nature. All other values set for simulation are same as for deterministic parameter case. Here, we record the mean value of estimates of 50 sensors as a vector in OMNeT++ for 600 data gathering cycles. The dynamic parameter was modeled as a discrete sinusoidal parameter given by sin(k/20) where k is the sensor network’s *k*th time step of measurement or in other words *k*th data gathering cycle. We track how the three different fusion schemes are able to track the varying parameter under different communication error rates as shown in Figure 10.

As it can be observed from results in Figure 10, all considered fusion methods follow the sinusoidal variation as the real system state for under low communication errors. That means for BER of 0.0001, there is less difference between the network mean estimates of different fusion techniques as mean estimates almost overlap on each other.

However, when BER is 0.05, it can be observed a deviation among the mean estimates three different fusion techniques. EBDKF and AVG fusion methods seem to have a higher deviation from the actual system state than the estimates from the proposed method. To prove the preceding argument, we calculated the network MSE for two different BER values for three fusion techniques as shown in Table 1.

As it is evident from the results summarized in Table 1, network MSE performance for dynamic parameter is such that EBDKF has highest accuracy for low BER and vice versa. Average method has least accuracy under low BER and moderate accuracy under high BER. The proposed method has moderate accuracy under low BER and highest accuracy under high BER. Therefore, the performance of the three considered fusion techniques in terms of accuracy for dynamic system parameter is similar to that of deterministic parameter. Therefore, the argument that proposed method outperforms both AVG and EBDKF under high communication errors can be derived for dynamic system parameter situation also. The reason for overlapping of estimates when BER of 0.0001 and the deviation when BER of 0.05 which was observed in Figure 10 can be explained using network MSE values summarized in Table 1. The MSE values of the three fusion techniques are very low and there is less difference between them when BER is 0.0001 and vice versa when BER is 0.05 is the reason for preceding observation.

#### 3.1.3. Evaluation of Complexity of Information Fusion

In chain-based WSNs, each node has only one child for AVG and proposed method. Thus, each node needs to combine only two data frames/packets during the fusion process for AVG and proposed methods. For EBDKF fusion technique, the average number of neighbors is two such that there will be 3 data frames/packets to combine in a single time for a node. Further, there are 3 batches of such data frames/packets per data gathering cycle. Therefore, the complexity is high in EBDKF. The number of CPU cycles of the microprocessor and the corresponding complexity $T_{A}$ for each fusion function is given in Table 2. We use Atmel Studio 7 to evaluate the complexity of the fusion function.

Single precision 32-bit floating-point data types are used in AVG, EBDKF and the proposed information fusion functions. The complexities of these functions are therefore high compared to simple information fusion functions such as OR and AND. The average method fusion function will require only two additions and one division. In the proposed statistical information fusion two additions, five divisions, and one multiplication are used. It was discussed in a previous section that the average number of neighbors for diffusion technique in a chain-based platform as two. For a single diffusion estimate, the Estimation based Diffusion Kalman Filtering will require 10 additions, 4 divisions and 8 multiplications. Thus, the EBDKF fusion function has a much higher computation complexity than the proposed method and AVG method as proved by results in Table 2. It can be identified that the complexity of diffusion technique has been 7 times complexity of average method whereas the proposed method’s complexity is only 1.97 times that of Average method for chain-based sensor networks.

#### 3.1.4. Network Lifetime

**Figure 11:**
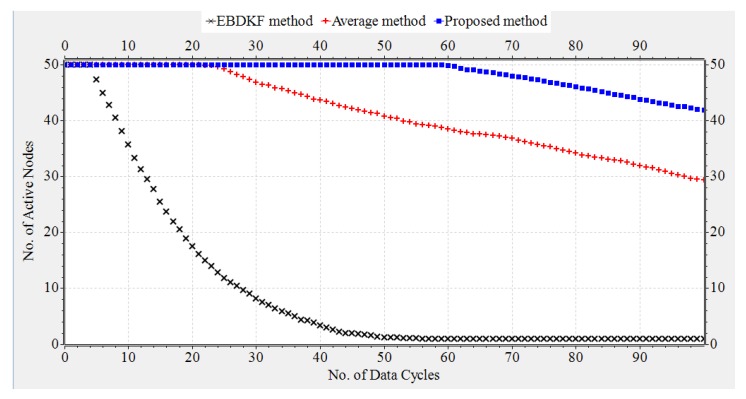
Network lifetime comparison with the proposed statistical, EBDKF and AVG information fusion methods when BER = 0.05 in a chain-based IoT platform.

The transmission energy, receiving energy, and circuit energy are the main determinants which differentiate network life of WSN s. In this section, we compare the network life time of a chain-based networks that separately uses AVG, EBDKF, and proposed statistical information fusion methods. A chain-based network with 50 nodes is considered for the simulation and they are spaced in equal distances. Previously in this paper, we proved that at BER of 0.05, when considering the accuracy of deterministic system parameter, AVG and EBDKF fusion techniques will require maximum retransmissions per hop per information frame/packet to be set to 4 and 1, respectively. Therefore, here we consider the network lifetime for such a situation in order to achieve an MSE less than 0.30. In that section, we neglected the communication burden due to sending of acknowledgment frames/packets. However, here we consider the energy loss due to acknowledgment frames/packets in a retransmission allowed network. Here, $b_{0}$ and $a_{0}$ are set to 0.3 and 0.1, respectively. We randomly assign values between 10 mJ to 50 mJ as initial energy levels for sensor nodes using a uniform distribution. We use Equation (10) to compute the energy consumption of each link. In Equation (10), we substitute $R_{d}=R_{a}=19.2$ kbps, $P_{t}=200\;\mathrm{mJ},P_{c}=P_{r}=100\;\mathrm{mJ}$ and $L_{d}=L_{a}=8$. Since Totaltransmissions=(TotalRetransmissions+1) maximum values for X and Y are set to 4, 2, and 1 for AVG, EBDKF, and proposed fusion methods, respectively. Further Voltage(V) and Current(I) values of the Central Processing Unit of wireless sensors are set to 5 V and 20 mA respectively to calculate the energy consumption due to computations using the fusion time values given in Table 2. We run extensive simulations and record number of total active nodes at each time step for 100 data gathering cycles and replicate for 50 times to obtain Figure 11, which depicts how the average total active nodes of the network change with time for 3 different fusion techniques.

We can see the improvement in the network life time when we employ the proposed statistical method by observing the results in Figure 11. When the proposed statistical method is employed, 59 data gathering cycles are possible prior to losing the first node in the network. But, in AVG method and EBDKF, the first node is died after 21 and four data gathering cycles, respectively. This experimental result also proves the mathematical derivation of total information transmission load comparison for three fusion techniques. There we mentioned that the information transmission of AVG and EBDKF as 3 and 9 times than proposed method for achieving network MSE less than 0.3. Further, we can observe that by 59 data gathering cycles, proposed method has lost none of its nodes, network with Average fusion had lost 28% of nodes and EBDKF has lost all of its nodes. When observing Table 2, it is very clear that highest computational efficiency belongs to Average method. However, as the proposed method does not send any retransmissions and acknowledgments, its overall energy efficiency is considerably higher than two other fusion techniques considered even though its computational complexity is moderate. The reason for EBDKF for producing least network lifetime is due to its very high computational complexity and very high number of retransmissions compared to other two fusion techniques. Therefore, the proposed method stands tall in terms of network lifetime so it is highly suitable to be used in wireless sensor networks used in smart grid environments having energy constraints.

#### 3.1.5. Network Latency

Using Equations (20) and (21), network latency of chain-based networks that separately uses AVG, EBDKF, and proposed statistical information fusion methods can be calculated. It can be assumed that for networks with proposed method and AVG that $t_{qu}=0$ as there is always only one frame/packet waiting to be served. However, in EBDKF, there can be multiple frames/packets waiting to be served so that frame/packet waiting time has to be considered and we assume $t_{qu}=0.1\;\mathrm{ms}$ We substitute R=20 kbps for Zig Bee, R=384 kbps for WCDMA, $l_{i}=50\;m$ for Zig Bee, $l_{i}=5000\;m$ for WCDMA, $c=3\times 10^{8}\;{\mathrm{ms}}^{-1}$ and P=20bytes for Zig Bee (1 byte payload, 19 bytes for data link layer header and trailer), P=41bytes for WCDMA (1 byte for payload, 40 bytes for TCP/IP header). *K* is set to 3, 1 and 0 for AVG, EBDKF and proposed fusion methods respectively to achieve similar MSE values (close to 0.3). Fusion times ($t_{fu}$) are obtained from Table 2. Table 3 summarizes the network latency values calculated in this manner.

As proved by the results in Table 3, when the proposed statistical method or AVG method is employed, the network latency is low when N low. However, on the other hand, EBDKF maintains a constant network latency independent of N. It can be shown that using results in Table 3 that AVG method and proposed method exceed the latency of EBDKF when N is greater than or equal to 5 and 14, respectively. Further, we can observe that the network latency of Average method is always almost 4 times that of that of proposed method in order to achieve similar accuracy.

When obtaining the results in Table 2, we assumed that inter-node link length is constant. As the proposed method is targeted to be employed in HANs and FANs, we expect the total length of the chain to be less than 500 m and 500 km for HANs and FANs, respectively. Therefore, the value of N will be restricted to 11 nodes and 101 nodes respectively for HANs and FANs as $l_{i}$ is 50 m and 5 km for Zig Bee used in HAN and WCDMA used in FAN. Therefore, the proposed method will have the lowest latency in a HAN so it is highly suitable to be used in a HAN compared to other two fusion methods standing tall in terms of network latency. For a HAN, EBDKF will have the highest latency when N is less than 5. When N is greater than 5, the AVG method will have the highest latency for a HAN using Zig Bee. When considering about FANs, the proposed method will maintain its dominance in terms of lowest latency until N is 14; but, after that, the latency of EBDKF becomes the lowest as the total length of the link exceeds 70 km. When considering about the Maximum tolerable latency of 300 ms for the SG applications intended in this context, the proposed method and diffusion technique will not violate the latency requirement for both HANs and FANs. That is because the proposed method will require at least 39 and 174 nodes for HANs and FANs, respectively, which are way above the limitations we assumed for N considering physical length of links for those networks. However, for the AVG method, it will require at least 11 nodes and 45 nodes to violate the latency requirement. Therefore, the AVG method will fail latency requirement in HAN by a small margin when N is 11 and it will also fail when the total length of the link in a FAN is high (exceeds 225 km in this case).

### 3.2. Simulations for Hierarchical Tree Structured Network

#### 3.2.1. Simulations for Data Accuracy of a Deterministic Constant (Slowly Varying) Parameter Having Bounded Sensor Noise

**Figure 12:**
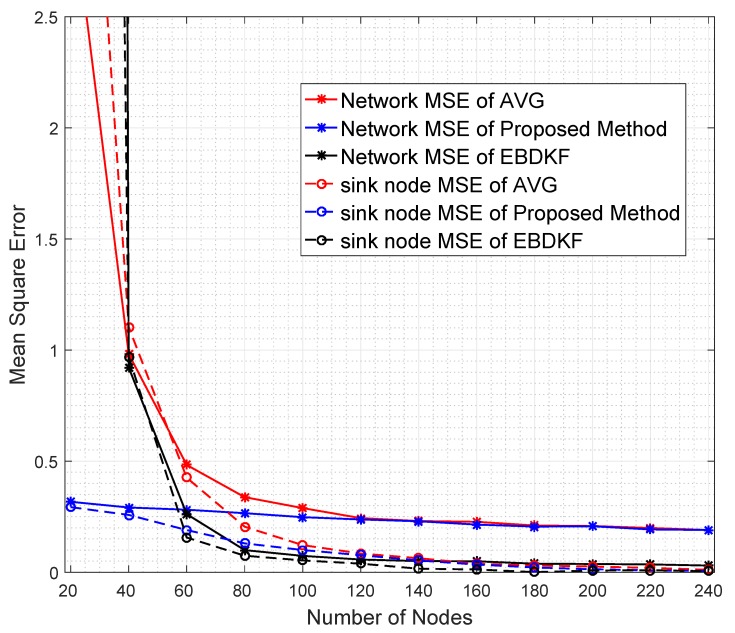
Accuracy comparison among the proposed statistical, AVG, and EBDKF information fusion methods in a tree-based IoT platform.

In this section, the mean square error of the network is analyzed by considering different network sizes that vary from 20 to 240 in steps of 20 nodes. The heterogeneous measurement noise variances are generated by setting $b_{0}=0.3$ and $a_{0}=0.1$. Figure 12 shows the network MSE comparison among three different fusion techniques for the considered network sizes.

When observing the results in Figure 12, it can be seen that the accuracy of the sink node and network using proposed method is always higher than that of conventional average method and the performance gap between these two approaches reduces with the number of nodes. The accuracy of those two methods become almost same when the number of nodes is greater than 120 for both network and the sink node. When we increase the number of nodes in the network, sensor nodes become closer to each other as the area in which sensor nodes are placed is maintained constant. Therefore, the average link distance is reduced, even though the individual link distances vary, making the resultant BER values of links in the network to be reduced. For example, when the number of nodes is increased from 20 to 240, the BER of a link is reduced from 0.2289 to 0.000002 following the shape similar to an inverse square law function. When the BER reduces, the performance gap of all three fusion methods for sink node’s accuracy approaches a very low value, but the accuracy is in the decreasing order of EBDKF, proposed method and average having a very low accuracy gap between each of them.

EBDKF fusion technique has the highest accuracy out of all the methods when the number of nodes is greater than 60 for both sink node and the network. The average BER value, when number of nodes is equal to 60, is 0.0173. Therefore, the proposed method shows highest accuracy when the average BER of a link is higher than 0.0173. When the number of Nodes is less than or equal to 40, i.e., when average BER of a link is greater than 0.0455, the EBDKF fusion’s accuracy degrades resulting least accuracy out of the three methods. The performance gap between the network and the sink node accuracy is very low. This can be explained as a result of both the network and the sink nodes having equivalent number (1.87 exactly as derived in following section on complexity) of average neighbors and since all nodes act as aggregating nodes. For chain-based network, the accuracy of sink node and network almost overlapped as seen in Figure 4. However, in contrast, for tree-based network the accuracy of the sink node had been always better than the network for proposed statistical method. The performance gap of the accuracy of network and sink node for proposed method reduces as the BER is increased. Lower accuracy of the network can be explained as a result of 47% (refer section on complexity evaluation) non-aggregating leaf nodes present in tree networks which do not use any fusion technique, but only transmit the measurement to immediate node. In tree network, the network MSE performance of the average method is higher than that of the chain-based network. The reason for this can be predicted as the increment of the average number of child nodes for a given node in tree-based network. In chain topology, the average number of child nodes is always equal to one and in tree it is almost two such that mean values fused in tree is more accurate than chain for average fusion. When comparing the accuracy of the average method for the tree network between its sink node at network, it can be identified that the accuracy of the sink node for low BER is higher than its network and vice versa. The performance of sink node and network has been similar when the number of nodes is around 55, that is BER of 0.0214. This phenomenon can also be explained as follows. The increment of BER does not affect the accuracy of the measurement in non-aggregating nodes for average method. Therefore, at low BER values, the accuracy of the network is low as the sensor noise of non-aggregating nodes have a significant impact. However, the accuracy of sink node is always affected by change in BER values as it fuses two already fused results in average for tree network. Therefore, under high communication errors, the sink node’s accuracy goes lower and the network accuracy is maintained higher by the non-aggregating sink nodes whose accuracy does not depend on BER s.

Further, there is no significant difference in network MSE of the proposed method between chain and tree sensor networks when comparing Figure 4 with Figure 12. However, for very low BER values (<10${}^{-5})$ in chain topology, the network MSE had been reduced to the level of EBDKF, whereas in tree topology, there is no significant improvement in the accuracy of the proposed method for such BER (220<nodes<240). The network MSE value of proposed method remain less than 0.31 for different number of sensor node combinations (all combinations of average BER values) in the considered range from 20 to 240 nodes. In comparison to these results, the MSE performance of EBDKF for very low BER values is higher for tree topology than chain topology and vice versa. This can be also explained as a result of increased branching in tree resulting higher number of neighbors for diffusion than chain topology. In chain, there are maximum of only two neighbors to exchange local and individual estimates. However, in tree topology as it will be proved in following section on complexity, there are in average three neighbors per non-leaf nodes for diffusion to take place So, under low BER values the diffusion process has increased the accuracy of network than chain by increased number of neighbors and at the same time it has been worsen under high BER values. These arguments can be further verified by analyzing the bit level accuracy under high and low communication errors as analyzed later in this section.

**Figure 13:**
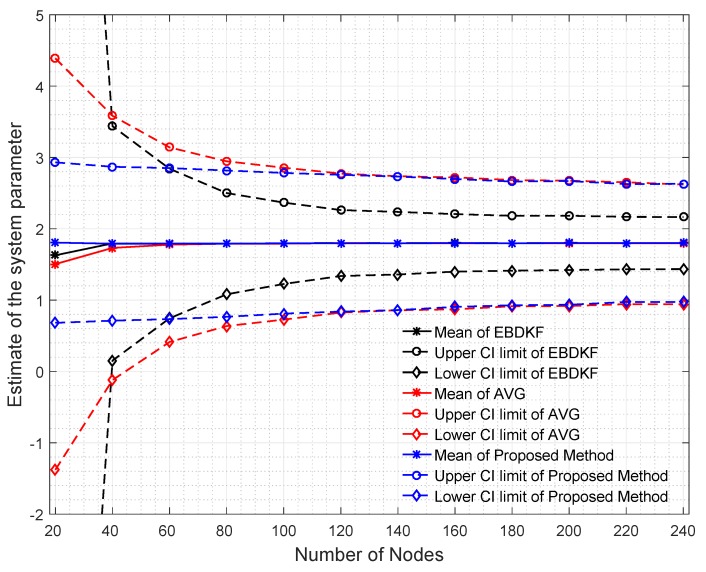
Network Mean estimate and 95% confidence interval limits for a true state of 1.80; compared among the proposed statistical, EBDKF, and AVG information fusion methods in a tree-based IoT platform for different number of sensor nodes.

To justify the network MSE values obtained for three different data fusion techniques in Figure 12, and to visually represent the mean estimate, we plot the mean estimate and 95% confidence limits for tree network as shown in Figure 13.

When analyzing the results obtained in Figure 13, it can be seen that for low BER values the network mean estimate is very close to the system state of 1.8 for all data fusion techniques. In these conditions, the 95% confidence interval is also narrow and its width is least in EBDKF and overlaps for Average and proposed method proving the previous result obtained for network MSE for low probability of communication errors. This is because we can say that when the confidence interval for the results is higher, so is the network mean square error. The mean estimate of the proposed method decreases slightly and remains very close to the real value of 1.8 when BER increases. But the mean value tends to deviate more from real value of 1.8 when BER increases for other two data fusion techniques where the EBDKF method having a moderate deviation and Average method having the highest deviation. The reason for observing high network MSE for high BER for average and EBDKF can be explained by looking at the increasing size of the 95% confidence interval for those two methods as evident from Figure 5. In contrast, the proposed method’s confidence interval is only slightly increased with increasing BER (decreasing nodes), so that it is showing least network MSE for high BER.

**Figure 14:**
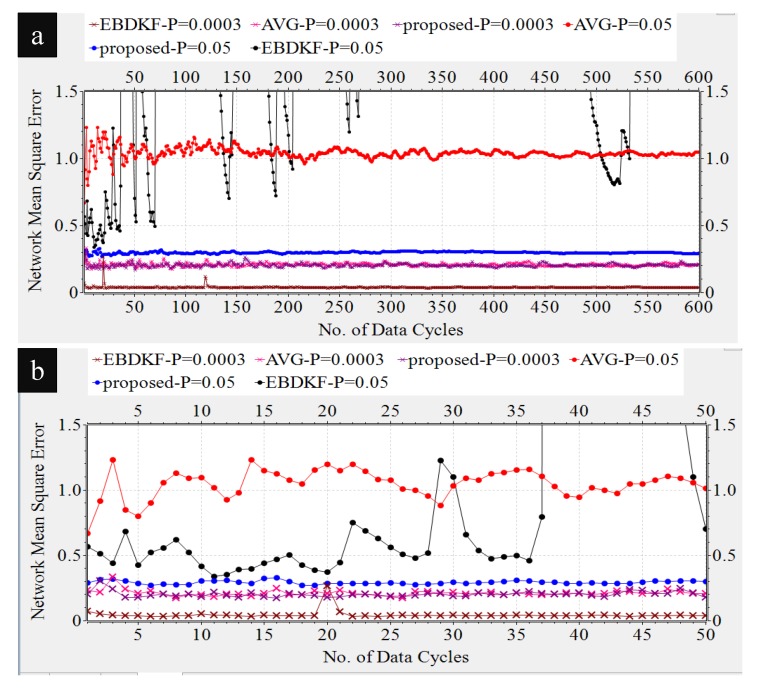
Network MSE vs. the number of data gathering cycles for BER of 0.0003 and 0.05 among the proposed statistical, AVG and EBDKF information fusion methods in a tree-based IoT platform: (**a**) for first 600 data gathering cycles and (**b**) for first 50 data gathering cycles.

It is very important to understand the impact of number of data gathering cycles on the accuracy of the estimate. Therefore, we plot the network MSE vs. the number of data gathering cycles as shown in Figure 14.

Average bit error rate of 0.05 corresponds to 38 nodes and that of 0.0003 corresponds to 200 nodes in tree structure. We selected these two BER values because when BER is 0.05, the accuracy of proposed method is higher than EBDKF and vice versa for BER of 0.0003 for 600 data gathering cycles as proven in Figure 12. Indeed, all the results generated so far corresponded to 600 data gathering cycles. As it is evident from Figure 14, the previous argument is unchanged for any data gathering cycle. Only the MSE gap is changed with data gathering cycles. For all fusion methods, the network MSE for high communication errors is always higher than that for low communication errors for any data gathering cycle.

The proposed method shows the least deviation and thus the highest stability on the network MSE against number of data gathering cycles. It shows higher fluctuations of network MSE for initial data gathering cycles (for up to about 50 cycles) for both BER s than when number of data cycles are much higher. Similar variation with data cycles is observed in AVG method, but its variations are higher than proposed method and it consumes a higher number of data cycles for the network MSE to get stabilized.

**Figure 15:**
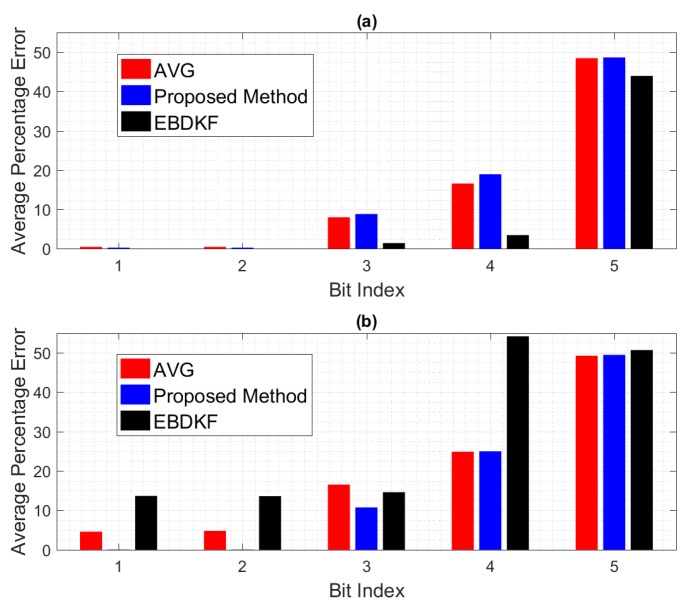
Bit level error performance comparison among the proposed statistical and the AVG information fusion method in a tree-based IoT platform when (**a**) BER = 0.005 and (**b**) BER = 0.05.

However, in contrast, the MSE performance with data cycles is different for data fusion scheme EBDKF under high communication errors. As proved by Figure 14, the variation of proposed method with data cycles even for high communication errors is almost same as that for low communication errors. When it comes to the diffusion technique, the network MSE is always lesser than 1.0 until first 28 data gathering cycles for BER of 0.05 case. After that, the network MSE value is often greater than 1.0. On the other hand, for low communication error scenario, EBDKF tend to be less accurate for initial data gathering cycles. That is because we can observe a big spike at 20 data cycles and a smaller spike at 120 and no spike after that with highly stable MSE for the graph of EBDKF for BER of 0.0003 in Figure 14. Therefore, it can be deduced that accuracy of diffusion technique is better for low data gathering cycles under high communication errors and vice versa. The proposed method has clearly outperformed both AVG and EBDKF under BER of 0.05 for any data gathering cycle as it was concluded for 600 data gathering cycles before. We further analyze the impact of BER on bit level error characteristics in Figure 15.

Two networks are simulated where network one has 38 nodes and network two has 98 nodes corresponding approximately to average BER of 0.05 and 0.005, respectively. These values are selected in order to have a fair comparison of the bit level accuracy among tree and chain topologies. When the proposed statistical information fusion method is employed, the most significant 4 bits are successfully recovered in both networks. The bit flipping in the most significant bits significantly change the accuracy of the fused value. In network two (BER = 0.005), the average error probabilities of most significant bits show lower values for both Average and proposed methods, in which the proposed method has a slightly lesser value. This is the reason to have a lower performance gap between those methods in Figure 12 and a low difference in confidence intervals in Figure 13, when network size is 98. In these conditions EBDKF show highest accuracy since its MSB error percentage is 0.0306. But when the BER is increased to 0.05 by network one having 38 nodes, a very high percentage increment of 4 significant bits of EBDKF is observed compared to BER of 0.005 case making its accuracy least whereas in Average method a relative lower increment in percentage error in significant bits are observed.

When comparing Figure 15 with Figure 6, to have a comparison among chain and tree sensor network accuracy wise performance, it can be seen that for Average method percentage increment of errors of significant bits for BER of 0.05 is lower in tree network topology confirming the argument that average fusion’s accuracy is higher in tree architecture than chain architecture. For both topologies, the proposed method shows almost similar percentage errors proving that accuracy level of the proposed method remains almost unchanged for chain and tree network topologies. Furthermore, under high communication errors, bit level percentage error values of EBDKF fused results for tree topology is higher than chain topology proving that accuracy is worse than chain topology for high BER values. Similarly, the converse of the preceding argument, that is bit level accuracy of chain is worse than tree networks of EBDKF for low BER values. The measurement errors have higher impact on the least significant bits. Therefore, we cannot see considerable improvement in the least significant bits 5, 6, and 7 for any of the fusion techniques considered. Therefore, we omitted them in Figure 6 and Figure 15.

**Figure 16:**
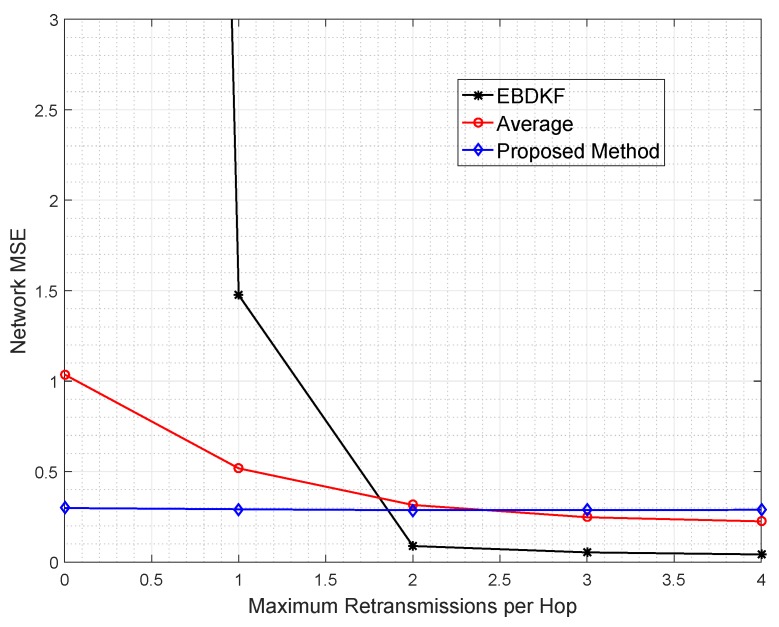
Network MSE comparison among the proposed statistical, AVG, and EBDKF information fusion methods in a retransmission allowed tree structured network when BER is 0.05.

It should be investigated how retransmissions affect the information accuracy in a tree structured network that has increased branching with respect to chain structure which does not have any branching. A theoretical upper bound for total data frame/packet transmission per data gathering cycle can be derived similar to Equation (38) derived for chain structure. We follow the same notation used for chain structure here also. We will derive in computation complexity section of fusion techniques for tree structure that 53% of nodes are aggregating nodes in fusion schemes which have unidirectional communication from source nodes to the sink node. Therefore, the total links of a tree structure is (k−1). Therefore, same upper bound in Equation (38) is valid for tree structure too. We extensively simulate the retransmission allowed tree structure for different fusion techniques by varying the maximum number of retransmissions allowed per hop to obtain the result given in Figure 16.

Number of nodes of the tree structure was selected as 38 corresponding to an average bit error rate of 0.05 in which the accuracy of the proposed method is highest as it was observed in Figure 12 and Figure 13. Here we have made the assumption that communication channel errors are not affecting on acknowledgment frames/packets so that an acknowledgment sent is always received. It can be clearly observed in Figure 16 that the final results for Average and proposed methods are similar to chain structure. That’s because proposed method does not show any significant improvement in accuracy with retransmissions and the AVG method needs maximum of three retransmissions per hop per data gathering cycle to achieve accuracy of the proposed method at a BER of 0.05. But, the EBDKF method needs to set maximum retransmission per hop to a value of 2 to overcome the accuracy of the proposed method. Therefore, L=0,2,3 in proposed method, EBDKF, Average fusion methods respectively in order to achieve a network MSE less than 0.3. As proved for chain structure, n=1,1,6 for AVG, proposed method and EBDKF respectively. Therefore, by substituting in Equation (39) for upper bound, $N_{proposed}=\left(k-1\right)$, $N_{AVG}=\left(4\right)*\left(K-1\right)$, $N_{EBDKF}=\left(18\right)*\left(K-1\right)$. Therefore, expected lifetime of the nodes using diffusion technique for tree structure is lesser than that for chain structure.

#### 3.2.2. Evaluation of Complexity of Information Fusion

**Figure 17:**
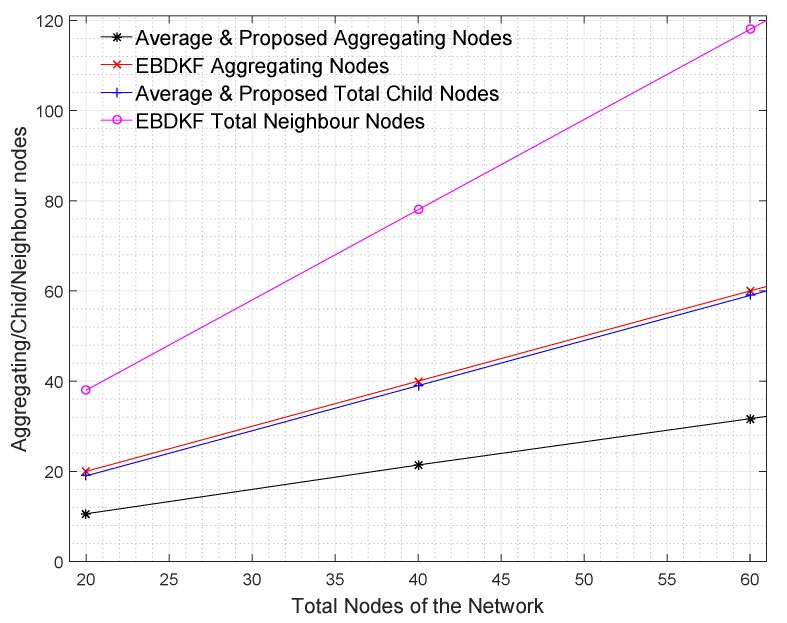
Average number of fusion nodes and total child nodes in tree-based IoT platform using maximum reliable connection method for different fusion techniques.

In tree-based network, multiple nodes can be connected to a fusing node. If the fusion node act as a small clusters head, the cluster head might have high computational complexity. Therefore, we analyze total number of fusion nodes in a tree network with minimum distance neighbor connection method that was specified earlier as depicted in Figure 17.

It can be observed in Figure 17 that approximately 53% of nodes act as aggregating nodes in networks using average and proposed method while all the nodes act as aggregating nodes in Diffusion Kalman Filtering techniques. As it can be observed in simulation results, the total number of child nodes in networks using average and proposed method for fusion has N−1 number of total child nodes, and a network using EBDKF will have 2∗(N−1) total child nodes where N is the total number of nodes. Therefore, the average number of child nodes per aggregating nodes of the network represented by $n_{child}$ for proposed and average fusion techniques will be as depicted in Equation (40).

(40) $$\begin{array}{cc} n_{child} & =\frac{\left(N-1\right)}{0.53N}\\ \; & =≋1.87 \end{array}$$

when *N* is high, $n_{child}$ value given in Equation (40) is close to 2. The leaf nodes for these fusion schemes have no child nodes. Now we will derive the $n_{child}$ for EBDKF as shown in Equation (41).

(41) $$\begin{array}{cc} n_{child} & =\frac{2*(N-1)}{N}\\ \; & =≋2 \end{array}$$

As it is evident from the Equation (41), a network employing EBDKF will also have average number of child nodes per aggregating nodes of the network almost equal to 2. Therefore, the average computational complexity of an aggregating node will be as if each aggregating node has two children for all of the data fusion techniques considered in this context. The number of leaf nodes for EBDKF are still 0.47(N−1) having one child per each node. Therefore, the total number of child nodes remaining for intermediate nodes will be as given in Equation (42).

(42) 2∗(N−1)−0.47∗(N−1)=1.53(N−1)

As there are 0.53(N−1) number of non-leaf nodes for EBDKF fusion, the average number of child nodes per non-leaf nodes ($n_{child_{non-leaf}}$) is given by Equation (43) as,

(43) $$\begin{array}{cc} n_{child_{non-leaf}} & =\frac{1.53(N-1)}{0.53(N-1)}\\ \; & =2.87 \end{array}$$

Therefore, as seen from Equation (43), the non-leaf nodes will have in average close to 3 child nodes. The root node is also a non-leaf node. Therefore, the root (sink) node will have in average 1.87 nodes that is approximately two nodes which is one lesser than a typical non-leaf node. So, the average number of child nodes for both the sink node and the network for a tree structured diffusion scheme is equivalent to two. Therefore, the average fusion complexity of the network and the fusion center (sink node) must be equivalents.

In chain-based WSNs, each node has only one child. Thus, each node needs to combine only two data frames/packets during the fusion process except the first node for AVG and proposed statistical method. But when it comes to the tree topology, an aggregating node needs to combine in average three data frames/packets in average considering the network. Two of them are information sent from the child nodes and the other is the self-measurement.

Now we will consider about the complexity of diffusion technique. The average number of neighbors of EBDKF for both chain and tree topology were proved to be two nodes in previous sections. Therefore, the fusion complexity of chain and tree topology for diffusion technique must be equivalent. The number of CPU cycles of the microprocessor and the corresponding complexity $T_{A}$ for each fusion function considering two children per aggregating node as argued above, are given in Table 4. We use Atmel Studio 7 to evaluate the complexity.

When considering the results obtained in Table 4, it can be seen that the average complexity of the proposed method is 3.25 times that of average, whereas the complexity of EBDKF has been 5.25 times that of average fusion complexity. When we compare the above result with the results obtained for complexity in information fusion functions for chain-based networks in Table 2, it can be seen that the average complexity of all fusion functions except EBDKF have been increased by 33.33%, 21.875% for average, proposed method respectively. Therefore, the increment of complexity of information fusion is least for Diffusion Kalman Filtering. In average, the AVG method will require three additions and one division, whereas the proposed statistical information fusion requires four additions, seven divisions, and one multiplication whereas the EBDKF fusion will require in average, 14 additions, four divisions and eight multiplications. Thus, still the complexity of diffusion technique is highest even though the topology is changed from chain to tree topology.

#### 3.2.3. Network Lifetime

**Figure 18:**
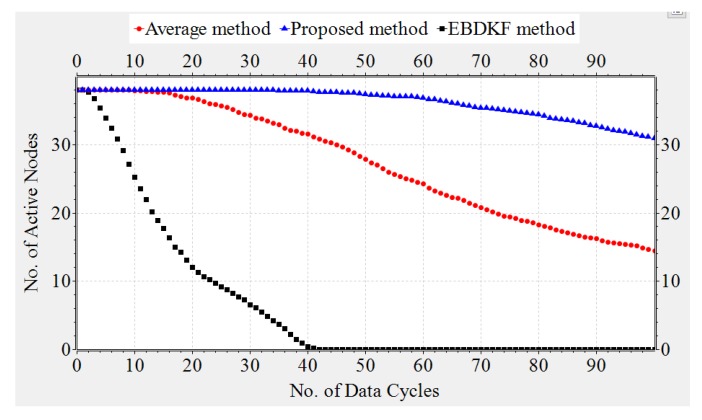
Network lifetime comparison for 3 information fusion methods in a tree-based IoT platform having 38 nodes.

In this section, we consider a network that consists of 38 nodes to analyze the network lifetime. The BER of a link is approximately equal to 0.05. The heterogeneous measurement noise variances are generated by setting $b_{0}=0.3$ and $a_{0}=0.1$. When AVG is used as fusion function, we allow maximum 3 retransmission attempts for each hop per data gathering cycle and when EBDKF is employed, 2 maximum retransmission attempts are set as justified in the previous section on retransmission allowed tree structure. The initial energy levels for nodes are assigned randomly between 10 mJ and 50 mJ using a uniform distribution. The total energy consumption of a link is determined using the Equation (10). All the parameters in Equation (10) are similar to the values we specified for the chain-based network simulations. Here, we monitor the number of nodes active in the network for different fusion techniques in order to achieve similar accuracy of the network. The information retransmission is therefore employed to improve the accuracy in EBDKF and AVG methods to achieve accuracy just higher than the proposed method. Figure 18 depicts the number of active nodes in the network when we use the proposed statistical, Diffusion Kalman Filtering and AVG methods in tree-based network at BER of 0.05. We run extensive simulations and record number of total active nodes at each time step for 100 data gathering cycles and replicate for 50 times.

As it can be clearly identified in Figure 18, with the proposed statistical method, the network can have 34 data gathering cycles using all nodes. This value goes down to 9 and 1 in AVG and EBDKF fusion techniques, respectively. The energy of the information and acknowledgment retransmissions becomes the dominant factor when deciding the network life time. The proposed method does not need to use any retransmissions to achieve better accuracy such that it can save energy on additional data transmission and reception, and acknowledgment transmission and reception. Therefore, the network lifetime is much longer than AVG and EBDKF methods. Further, it can be observed that the rate of losing active nodes in EBDKF method is higher than both AVG and proposed methods. It can be observed that after 42 data gathering cycles when network using EBDKF fusion loses all of the nodes, network with proposed method and AVG will lose only 2.6% and 15.8% of nodes respectively. Therefore, the network lifetime in decreasing order is proposed method, AVG and EBDKF respectively.

#### 3.2.4. Network Latency

A similar analysis on network latency for tree structure can be performed as it was done for chain-based networks. The sink node can be considered to be in the center of a circle covered by other nodes of the network where the maximum length of the link is the radius of that circle. Therefore, N is the number of nodes in such a radius. To cover a similar area that was covered for chain, we can assume that maximum number of nodes in longest link of tree network as half of maximum nodes that can exist in chain network. That is, N is 6 and 51 for HANs and FANs, respectively, thus expecting the total length of the communication between furthest source node and sink node to be less than 250 m and 250 km for HANs and FANs, respectively. Therefore, maximum expected latency of both AVG and proposed methods reduce by almost half that of latency expected for chain-based networks. Therefore, for tree-based networks, conclusions on network latency of the proposed method and EBDKF are same as that of chain-based networks. But in tree structured networks the AVG method can also satisfy the latency requirement in HANs and violates the latency requirement of FANs when N is greater than or equal to 45 as the maximum length of communication substantially has been reduced compared to chain-based networks.

### 3.3. Simulation for Data Accuracy in Randomly Placed Non-Hierarchical Bidirectional Graph

#### 3.3.1. Simulations for Data Accuracy of a Deterministic Constant (Slowly Varying) Parameter Having Bounded Sensor Noise

**Figure 19:**
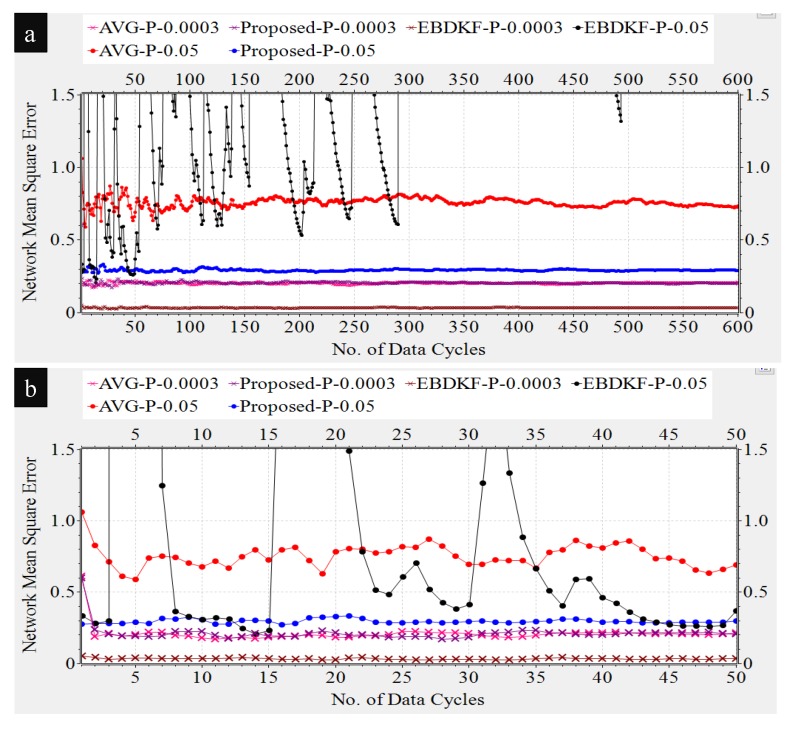
Accuracy comparison with Data gathering cycles for BER s of 0.0003 and 0.05 (**a**): For first 600 Data gathering cycles (**b**): For first 50 Data gathering cycles.

The link distance is unchanged and the BER of a link is varied by varying the path loss exponent to obtain the average bit error rate of a link in the network using analytical model given in [56]. First, a network MSE comparison of data fusion techniques with data cycles is obtained as shown in Figure 19.

Bit error rates of 0.05 and 0.0003 were selected for comparison as it was done for tree structure. Only the MSE gap is changed with data gathering cycles. For all fusion methods, the network MSE for high communication errors is always higher than that for low communication errors for any data gathering cycle.

The proposed method shows the least deviation and thus the highest stability on the network MSE against number of data gathering cycles. It shows higher fluctuations of network MSE for initial data gathering cycles (for up to about 40 cycles) for both BER s than when number of data cycles are much higher. Similar variation with data cycles is observed in AVG method but its variations are higher than proposed method and consume a greater number of data cycles for the network MSE to get stabilized.

However, in contrast, the MSE performance with data cycles is different for data fusion scheme EBDKF under high communication errors. As proved by Figure 19, the variation of proposed method with data cycles even for high communication errors is almost same as that for low communication errors. When it comes to the diffusion technique, the network MSE is most of the time lesser than 1.0 for first 50 data gathering cycles for BER of 0.05 case. After that, the network MSE value is often greater than 1.0. On the other hand, for low communication error scenario, EBDKF tend to be less accurate for initial data gathering cycles; this is because we can observe smaller fluctuations in MSE within first 50 data cycles and highly stable MSE for data cycles closer to 600 of EBDKF for BER of 0.0003 in Figure 19. Therefore, it can be deduced that accuracy of diffusion technique is better for low data gathering cycles under high communication errors and vice versa. The proposed method has clearly outperformed both AVG and EBDKF under BER of 0.05 for any data gathering cycle except for data cycles 13 to 15 and 45 to 49.

**Figure 20:**
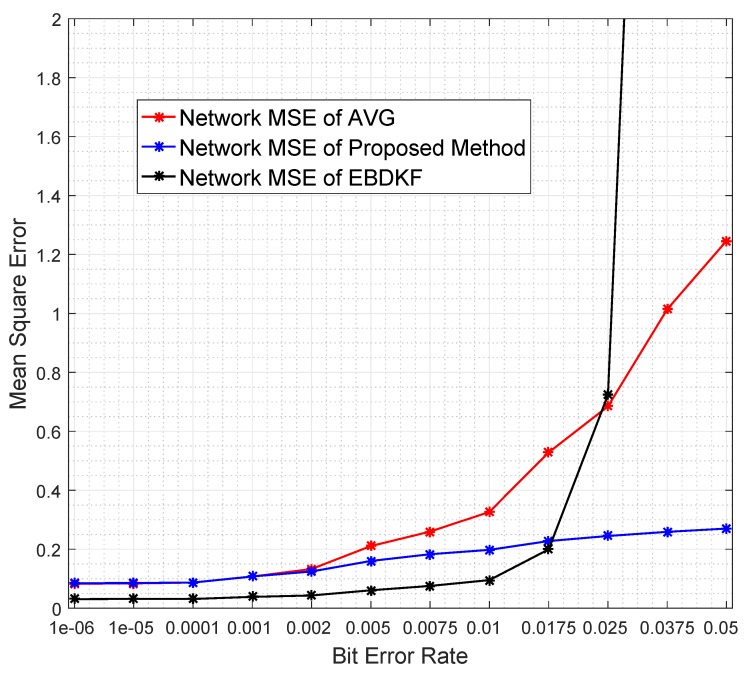
Accuracy comparison of 3 different fusion techniques for an ad hoc-type network.

We plot the network MSE versus the communication errors for AVG, proposed method and EBDKF for the previously discussed unstructured sensor network as shown in Figure 20.

The result obtained in Figure 20 is very similar to the result obtained for tree structure seen in Figure 12. Further, conclusions derived based on Figure 19 are also very similar to those derived on tree structure. Therefore, similar conclusions can be derived on the accuracy of unstructured network for three different fusion techniques. Therefore, the accuracy-wise performance of the information fusion techniques can be expected to be almost unchanged when the network gets nonhierarchical from hierarchical.

#### 3.3.2. Evaluation of Complexity of Information Fusion

In this section, we will generalize the complexity of information fusion in an unstructured network which retransmissions are not allowed. Let “*j*” be the average number of neighbors of the unstructured network. In chain and tree-based networks, the value of *j* was approximately 1 and 2, respectively. However, for unstructured network *j* can be any value depending on the instance of the network. For instance, the network that we used for simulation in Figure 3 has j=3.2. j can be obtained using Equation (44).

(44) $$\begin{array}{c} j=\sum_{i=1}^{N}\frac{\left(degree-1\right)}{N} \end{array}$$

where *i* is the *i*th node and *N* is the total number of nodes. In other words, each node of the network in average needs to fuse *j* number of frames/packets received from neighbors per data gathering cycle for AVG and proposed methods whereas EBDKF will receive 3*j* frames/packets. Table 5 summarizes the complexity of information fusion when *j* varies in an ad hoc-type Network.

As it can be observed from the generalized results in Table 5, the average fusion complexity will be in decreasing order of EBDKF, proposed method and AVG.

#### 3.3.3. Network Lifetime

In this section, we investigate the network lifetime of a bidirectional graph in which retransmissions are not allowed. When retransmissions do not occur, the energy on acknowledgment frame/packet transmission and reception will be zero. Therefore, the Equations (7) and (8) will reduce to Equations (45) and (46), respectively.

(45) $$E_{TX}=X\left(P_{c}+P_{t}/\eta \right)\left(L_{d}/R_{d}\right))$$

(46) $$E_{RX}=X\left(P_{r}L_{d}/R_{d}\right)$$

**Figure 21:**
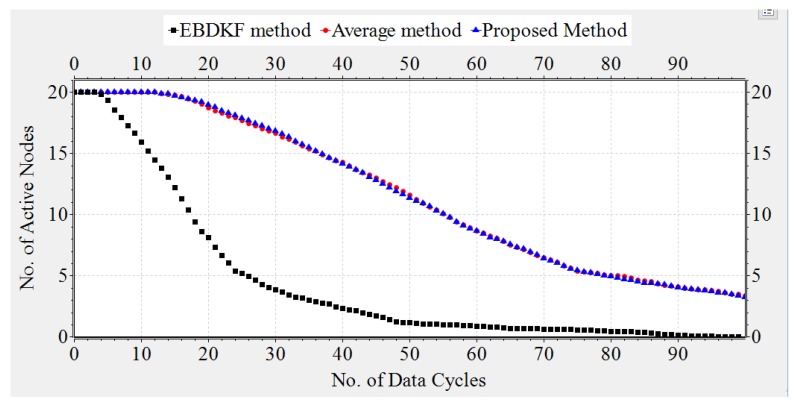
Network Lifetime comparison of three different fusion techniques for an ad hoc-type network.

As proved in the complexity section, X=j,j,3j for Average, Proposed, and EBDKF fusion techniques, respectively. $P_{c}$ also varies depending on the complexity of the information fusion technique. Since the complexity of the three fusion techniques increase in the order of Average, Proposed and EBDKF; $P_{c}$ is also expected to vary in the same manner. By arguing in this manner and using Equations (45) and (46); it can be derived that for an ad hoc network which retransmissions are not allowed, the network lifetime in decreasing order is Average, Proposed method and EBDKF. We simulate the network given in Figure 3 where *j* is approximately 3 to prove the preceding argument as shown in Figure 21. For this experiment also, we use Atmel studio 7 to evaluate the processing cycles to obtain the CPU time as shown in Table 6.

It can be observed that a similar performance with respect to network life time occurs when we employ the proposed statistical method and AVG method by observing the results in Figure 11. When the proposed statistical method or AVG is employed, 12 data gathering cycles are possible prior to losing the first node in the network. This value is much lower with respect to 59 obtained for proposed method in chain-based network as it was observed in Figure 11. The reason for the difference occurs as in the unstructured network considered, a node in average broadcasts 3 data frames/packets in a data cycle while in chain-based network it was only one. When observing Table 6, it can be observed that the fusion complexity of proposed method is three times that of average method. In spite of that, the network lifetime has been similar because of the equal j values and no retransmissions are allowed. The energy consumed for data fusion has been less significant with respect to the energy consumption for transmissions. That is because when combined with the fact that energy for transmitting a single bit is quite expensive than computing a single instruction, the energy consumption for the computational complexity of the proposed statistical method can be neglected.

On the other hand, in EBDKF, the first node is died after four data gathering cycles. Further, we can observe that by 97 data gathering cycles, networks employing Average fusion and proposed method had lost 85% of nodes and EBDKF has lost all of its nodes. When observing Table 6 it is very clear that highest computational consumption for fusion belongs to EBDKF method and j=9 are the reasons for having least network lifetime out of fusion techniques. Therefore, the simulation results agree with the theoretical predictions that we made earlier.

#### 3.3.4. Network Latency

Using Equations (21) and (22), network latency of bidirectional ad hoc networks that separately uses AVG, EBDKF, and proposed statistical information fusion methods can be calculated. All parameters used in analysis of network latency for chain-based networks are substituted in Equations (21) and (22) except for parameters given in following text. As there are only one sixth of packets or frames/packets waiting to be served, it can be assumed that for networks with proposed method and AVG that $t_{qu}=0.1/6\;ms$. K is kept as a generic value without setting a value. Fusion times ($t_{fu}$) are obtained from Table 6. Table 3 summarizes the network latency values calculated in this manner.

It can be observed from the results in Table 7 that when comparing the total latency value of latency due to information fusion ($t_{fu}$) can be neglected. None of the fusion techniques violate the latency requirement for both HANs and FANs up to K = 6. If K values are equal, the fusion methods in the increasing order of network latency will be Average method, proposed method and EBDKF. AVG method very slightly outperforms the proposed method by 7.5 μs such that network latency of proposed method is almost equal to that of AVG in network with same K values. However, as it was described in sensor networks using chain and tree structured networks, to achieve the same level of low accuracy as the proposed method under high BER conditions that exist in Smart Grid environments, retransmissions have to be allowed in networks deploying AVG and EBDKF. In that case, Proposed method will outperform both other fusion methods.

## 4. Conclusions

Measurement errors, quantization errors, and transmission errors are the three major sources of errors that affect the information fusion performance of IoT platforms in smart grids. We incorporate the statistical properties of these errors to information fusion to improve the information accuracy. The proposed statistical information fusion function not only shows highest information accuracy on popular information fusion structures, namely, chain and tree, but also in ad hoc networks under high communication errors (BER>0.0175) when compared with AVG and EBDKF fusion methods. Further, for chain network EBDKF outperformed the proposed method’s accuracy for $10^{-5}<BER<10^{-2}$ and for tree structure and ad hoc-type networks when BER<0.017. The accuracy of EBDKF fusion method varies having a high deviation for initial data gathering cycles under high communication errors while network MSE performance of all fusion techniques are considerably stable under low channel errors. The computational complexity of fusion techniques is always in the increasing order of AVG, proposed and EBDKF methods, respectively. When retransmissions are not allowed, simulation results prove that the proposed method achieves a network lifetime similar to AVG method and outperform the diffusion techniques for any BER. Fusion functions use different techniques such as retransmissions and redundant node deployment to improve the information accuracy under high communication errors. As the proposed statistical approach does not require these supporting techniques, it not only saves a large amount of energy and results in a longest network life time, but also leads to least network latency in HANs for both structured and unstructured networks and FANs in ad hoc-type networks outperforming both AVG and EBDKF in retransmission allowed networks. Further, the proposed method satisfies the maximum tolerable network latency requirement of 300 ms for SG applications targeted in this context for both FANs and WANs in all types of networks. When proposed method is used in structured FANs such as chain and tree, EBDKF outperformed the latency of proposed method when the link length is high. Therefore, under smart grid communication conditions, the proposed method is having the highest accuracy and network lifetime for all chain, tree and graph networks. When the effect of all the factors such as information accuracy, fusion complexity, latency and energy efficiency are combined, even under low communication errors, the overall performance of the proposed method can be considered to be higher than other fusion techniques considered in this context.

## Figures and Tables

**Table 1:** Network MSE for a dynamic system parameter for different fusion functions under different BER.

Fusion Function	MSE for BER = 0.00001	MSE for BER = 0.05
EBDKF	0.0428	266.5
AVG	0.1162	2.0107
Proposed method	0.0702	0.3019

**Table 2:** CPU Overhead of Different Information Fusion Functions for chain-based Network.

Fusion Function	CPU Cycles	Time (($T_{A}$)(μs))
Average	36	2.25
Proposed Method	128	8
EBDKF	252	15.75

**Table 3:** Network Latency of different information fusion functions for chain-based network.

Fusion Function	Latency for Zig Bee (ms)	Latency for WCDMA (ms)
Average	32.004(N−1)	6.97(N−1)
Proposed Method	8.008(N−1)	1.74(N−1)
EBDKF	98.42	23.316

**Table 4:** CPU overhead of different information fusion functions for hierarchical network.

Fusion Function	CPU Cycles	Time (($T_{A}$)(μs))
Average	48	3
Proposed method	156	9.75
EBDKF	252	15.75

**Table 5:** Average fusion complexity per node for different information fusion functions in a bidirectional graph.

*j*	Fusion Function	Complexity of Fusion
1	Average	2 Additions, 1 division
1	Proposed method	2 Additions, 5 divisions, 1 multiplication
1	EBDKF	6 Additions, 4 divisions, 8 multiplications
2	Average	3 Additions, 1 division
2	Proposed method	4 Additions, 7 divisions, 1 multiplication
2	EBDKF	10 Additions, 4 divisions, 8 multiplications
3	Average	4 Additions, 1 division
3	Proposed Method	6 Additions, 9 divisions, 1 multiplication
3	EBDKF	14 Additions, 4 divisions, 8 multiplications
n	Average	(n+1) Additions, 1 division
n	Proposed Method	2n Additions, 3+2n divisions, 1 multiplication
n	EBDKF	(4n+2) Additions, 4 divisions, 8 multiplications

**Table 6:** CPU overhead of different information fusion functions for randomly placed non-hierarchical graph.

Fusion Function	CPU Cycles	Time (($T_{A}$)(μs))
Average	60	3.75
Proposed method	180	11.25
EBDKF	1387	86.6875

**Table 7:** Network latency of different information fusion functions for non-hierarchical sensor network.

Fusion Function	Latency for Zig Bee (ms)	Latency for WCDMA (ms)
Average	8.034(K + 1) + 0.00375	1.775(K + 1) + 0.00375
Proposed Method	8.034(K + 1) + 0.01125	1.775(K + 1) + 0.01125
EBDKF	49.202(K + 1) + 0.0867	11.65(K + 1) + 0.0867

## Appendix A. Proof of the Upper Bound (17)

We denote information fusion node as *K* and a child node as *i* where i=1,2,…,K−1. We substitute ${\sigma^{2}}_{r_{i}^{{}^{\prime }}}=\sigma_{i}^{2}$, ${\sigma^{2}}_{s_{K}}=\sigma_{K}^{2}$ and $e_{i}=K-1$ in Equation (34). Then, we have to prove,

(A1) $${\left(\sum_{i=1}^{K}1/{\sigma^{2}}_{i}\right)}^{-1}\leq 1/{\left(K\right)}^{2}\sum_{i=1}^{K}{\sigma^{2}}_{i}$$

When *K* = 2,

(A2) $$\begin{array}{cc} {\left(\sum_{i=1}^{K}1/{\sigma^{2}}_{i}\right)}^{-1} & ={\left(1/{\sigma^{2}}_{1}+1/{\sigma^{2}}_{2}\right)}^{-1}\\ \; & \leq {\left({\sigma^{2}}_{1}+{\sigma^{2}}_{2}\right)}^{2}/\left(4\left({\sigma^{2}}_{1}+{\sigma^{2}}_{2}\right)\right)\\ \; & \leq \left({\sigma^{2}}_{1}/4\right)+\left({\sigma^{2}}_{2}/4\right) \end{array}$$

The inequality in the second step is due to the fact that $2{\sigma^{2}}^{1}{\sigma^{2}}_{2}\leq \left({\sigma^{2}}_{1}+{\sigma^{2}}_{2}\right)$ Therefore, Equation (A1) holds. Now assume the result in Equation (A1) holds for any i=n≥2. We have

(A3) $$\begin{array}{cc} {\left(\sum_{i=1}^{n}1/{\sigma^{2}}_{i}\right)}^{-1} & =1/{\left(n\right)}^{2}\sum_{i=1}^{n}{\sigma^{2}}_{i}\\ n^{2} & \leq \sum_{i=1}^{n}{\sigma^{2}}_{i}\sum_{i=1}^{n}{\left({\sigma^{2}}_{i}\right)}^{-1} \end{array}$$

Add another child to the node K where variance of a child node is $\sigma_{n+1}^{2}$. Then, we have the right side of above inequality. We can also write,

(A4) $$\begin{array}{c} n^{2}+\sum_{i=1}^{n}{\sigma_{i}}^{2}{\left({\sigma^{2}}_{n+1}\right)}^{-1}+{\sigma^{2}}_{n+1}\sum_{i=1}^{n}{\left({\sigma^{2}}_{i}\right)}^{-1}+1\\ \leq \left(\sum_{i=1}^{n}{\sigma_{i}}^{2}\left({\sigma_{n+1}}^{2}\right)\right)\left(\sum_{i=1}^{n}{\left({\sigma_{i}}^{2}\right)}^{-1}{\left({\sigma^{2}}_{n+1}\right)}^{-1}\right) \end{array}$$

Now consider,

(A5) $$\begin{array}{cc} \; & \sum_{i=1}^{n}{\sigma_{i}}^{2}{\left({\sigma_{n+1}}^{2}\right)}^{-1}+{\sigma_{n+1}}^{2}\sum_{i=1}^{n}{\left({\sigma^{2}}_{i}\right)}^{-1}+2n\\ \; & =\sum_{i=1}^{n}({\sigma_{i}}^{2}{\left({\sigma_{n+1}}^{2}\right)}^{-1}+{\sigma_{n+1}}^{2}{\left({\sigma^{2}}_{i}\right)}^{-1}+2)\\ \; & =\sum_{i=1}^{n}\left({\sigma_{i}}^{2}\left({\sigma^{2}}_{n+1}\right)\right){\left({\sigma^{2}}_{n+1}\right)}^{-1}+{\left({\sigma_{i}}^{2}\right)}^{-1})\\ \; & \geq 4n \end{array}$$

The above inequality is based on the result of Equation (A2). Now, we can write Equation (A4) as,

(A6) $$\begin{array}{cc} \; & {\left(n+1\right)}^{2}\leq \sum_{k=1}^{n+1}{\sigma_{i}}^{2}\sum_{i=1}^{n+1}{\left({\sigma_{i}}^{2}\right)}^{-1}\\ \; & (\sum_{i=1}^{n+1}1/{\sigma_{i}}^{2}{)}^{-1}\leq 1/\left(n+1\right)\sum_{i=1}^{n+1}{\sigma_{i}}^{2} \end{array}$$

This concludes the proof by mathematical induction.
